# Effect of soil additives on biogeochemistry of ultramafic soils—an experimental approach with *Brassica napus* L

**DOI:** 10.1007/s10661-024-12897-4

**Published:** 2024-07-17

**Authors:** Artur Pędziwiatr, Jakub Kierczak, Anna Potysz, Anna Pietranik

**Affiliations:** 1 Department, of Soil Science, Institute of Agriculture, Warsaw, University of Life Sciences, Nowoursynowska Str. 159, B.37, 02-787 Warsaw, Poland; 2 Department of Experimental Petrology, Faculty of Earth Sciences and Environmental Management, Institute of Geological Sciences, University of Wrocław, Maxa Borna Str. 9, 50-204 Wrocław, Poland

**Keywords:** Metallic elements availability, Spinel group minerals, Weathering, Serpentinite, Safe crop

## Abstract

Ultramafic soils are characterized by low productivity due to the deficiency of macroelements and high content of Ni, Cr, and Co. Incorporation of ultramafic soils for agricultural and food production involves the use of fertilizers. Therefore, this study aims to find the soil additive that decreases the metallic elements uptake by plant using *Brassica napus* as an example. In this study, we evaluate the effect of manure (0.5 g N/kg of soil), humic acids (1 g of Rosahumus/1 dm^3^ H_2_O; 44% C), KNO_3_ (0.13 g K/kg of soil), lime (12.5 g/kg of soil), (NH_4_)_2_SO_4_ (0.15 g N/kg of soil), and Ca(H_2_PO_4_)_2_) (0.07 g P/kg of soil) on the phytoavailability of metallic elements. The effect of soil additives on metallic elements uptake by *Brassica napus* was studied in a pot experiment executed in triplicates. Statistical analysis was applied to compare the effects of additives in ultramafic soil on plant chemical composition relative to control unfertilized ultramafic soil (one-way ANOVA and Kruskal–Wallis test). The study shows that in almost all treatments, metallic elements content (Ni, Cr, Co, Al, Fe, Mn) is higher in roots compared to the aboveground parts of *Brassica napus* except for (NH_4_)_2_SO_4_, in which the mechanism of Mn accumulation is opposite. The main differences between the treatments are observed for the buffer properties of soil and the accumulation of specific metals by studied plants. The soils with the addition of lime and manure have the highest buffer properties in acidic conditions (4.9-fold and 2.1-fold increase relative to control soil, respectively), whereas the soil with (NH_4_)_2_SO_4_ has the lowest effect (0.8-fold decrease relative to control soil). Also, the addition of manure increases the biomass of aboveground parts of *B. napus* (3.4-fold increase) and decreases the accumulation of Ni (0.6-fold decrease) compared to plants cultivated in the control soil. On the contrary, the addition of (NH_4_)_2_SO_4_ noticeably increases the accumulation of Ni, Co, Mn, and Al in aboveground parts of *B. napus* (3.2-fold, 18.2-fold, 11.2-fold, and 1.6-fold, respectively) compared to plant grown in control soil, whereas the humic acids increase the accumulation of Cr in roots (1.6-fold increase). Therefore, this study shows that manure is a promising fertilizer in agricultural practices in ultramafic soil, whereas (NH_4_)_2_SO_4_ and humic acids must not be used in ultramafic areas.

**Supplementary Information:**

The online version contains supplementary material available at 10.1007/s10661-024-12897-4.

## Introduction

Currently, the European Union is working on implementing a law on soil monitoring, where habitats with naturally high content of metallic elements that are included in Annex I of Council Directive 92/43/EEC shall be protected (Document 1). Nowadays, it can be observed that some habitats with soils naturally enriched metallic elements are incorporated into food production. Ultramafic soil is an example of soil that is naturally rich in metallic elements. It is a common name for soils derived from ultramafic rocks (peridotites, pyroxenites, and serpentinites; Kierczak et al., 2021). The major characteristic traits of ultramafic soils are (a) relatively high Ni, Cr, Co, and Mn content; (b) low availability of Ca relative to Mg; and (c) deficiency in essential macroelements, i.e., P and K (Baumeister et al. 2015; Kazakou et al., 2008; Weber 1981; Rodríguez-Seijo et al. 2017). Chemical composition of ultramafic soils (high content of Ni, Cr, and Co; low Ca/Mg ratio, and deficiency of macroelements) makes these soils unfavorable for practical use and potentially hazardous for human and animal health. The risk for humans and also for animals is especially high, when plants intended for food production are cultivated in ultramafic soils, even if the properties suggest that these soils should not be used for cultivation. The analysis of chemical composition of crops from ultramafic soils confirms the problem of increased uptake of metallic elements by plants growing in ultramafic soils. For example, Hseu and Lai (2017) noticed an elevated content of Ni in brown and polished rice growing in ultramafic soils in Taiwan (up to 4.53 and 5.54 mg kg^−1^ DW, respectively). Their study suggested that, based on the tolerable level of Ni intake for adults recommended by the World Health Organization, consumption of this rice could result in excessive Ni intake in humans. Similar problems were also noted for agricultural ultramafic soils located in India, Poland, Portugal, Spain, and Taiwan (Fernández et al., 1999; Hseu & Lai, 2017; Kierczak et al., 2021; Kumar & Maiti, 2014; Ramalhosa et al., 2019). Surely, the research in many countries has allowed to increase the knowledge on the negative effects of ultramafic soils on plants that are used by humans. However, the continuous increase in the human population leads to an increased demand for food. The decreasing area of soil suitable for cultivation means that even those soils with unfavorable properties, including ultramafic soils, are being incorporated into agricultural production. Therefore, the cultivation of such soils requires special attention. For example, the effect of fertilizers, not only on plant yield but also on the chemical composition of plants, has to be considered. It is important for plants to reduce the bioavailability of metallic elements from ultramafic soil. For example, the mobility of metallic elements in ultramafic soil can be decreased by adding biochar to soils (Galey et al., 2017). However, biochar can reduce the enzymatic activity in ultramafic soils. The impact of ultramafic soils on a variety of environmental parts can also be mitigated by the phytoextraction of metallic elements from soils (clean-up of soils by plants). For example, some studies revealed that the phytoextraction of Ni by Ni-hyperaccumulators can be increased due to biomass increase after the addition of compost to ultramafic soils (Álvarez-López et al., 2016). What is important, the decrease of Ni mobility in soils was observed at the same time. The studies with compost showed that the phytoextraction of metallic elements from soils can be increased. Therefore, the ultramafic soils and heaps with ultramafic mining wastes can be depleted in metallic elements making the soil more useful. Furthermore, the use of compost falls into the concept of circular economy.

The aim of our study was to find a soil additive that is the most beneficial for ultramafic soils. We focused on three aspects: (1) decreasing the phytoavailability of Ni, Cr, Co, Mn, Fe, and Al; (2) increasing the biomass of plants; (3) increasing the availability of beneficial elements (P, N). Therefore, in this study, we assessed the effect of six soil additives on the phytoavailability of Ni, Cr, Co, Mn, Al, and Fe for *Brassica napus* growing in ultramafic soil. The analyzed soil additives were selected due to their widespread use in agriculture and because some of the selected soil additives are sources of C, an element which is relatively scarce in Europe’s soils. To determine the role of soil additives in limiting the phytoavailability of selected metallic elements, we used an approach which combined measurement of the buffer properties of soils, direct analysis of soil solutions collected from pots, and analysis of the chemical composition of plants. Therefore, this study can help improve the management of arable ultramafic soils, leading to the production of higher yields of safer food compared to unfertilized ultramafic soils. Furthermore, this study can increase knowledge about the influence of commercial humic substances on plant growth in ultramafic soils. This is important because there is increasing interest in using new soil fertilization techniques, which include commercial humic substances. It should be also emphasized that this study fills the research gap concerning the effect of fertilizers on the environmental stability of spinel-group minerals—principal Cr-carriers in ultramafic soils, which are considered to be resistant to weathering.

## Materials and methods

### Materials

Ultramafic soils for the pot experiment were collected in the Jordanów site (SW Poland) from depths of up to 25 cm in an arable ultramafic field. A detailed description of the study area can be found elsewhere (Kierczak et al., 2016; Pędziwiatr et al., 2018). Ultramafic soils in this area are represented by Eutric Skeletic Cambisol (Loamic) soils, and these soils have developed on proper serpentinite. During the field studies, seeds of *B. napus* were collected for the pot experiment to reflect local conditions. *B. napus* was selected for the pot experiment because it is often cultivated in ultramafic soils. Furthermore, *B. napus* is used for oil production. Studied plant species is also used for livestock feed due to the relatively high content of protein. Furthermore, plowing of arable field with straw of *B. napus* is able to fertilize the soil.

### Pot experiment

The experiment was carried out in a greenhouse at the Warsaw University of Life Sciences (air conditions are presented in Appendix 1). The pots were filled with 2 kg of soils per pot. The experimental variants were executed in triplicates. The following treatments were prepared: (a) control (only ultramafic soil; T1), (b) manure (ultramafic soil with granulated cow manure; T2), (c) rosahumus (ultramafic soil watered with humic acids trademarked as Rosahumus; T3), (d) KNO_3_ (ultramafic soil with KNO_3_; T4), (e) lime (ultramafic soil with lime; CaCO_3_ form; T5), (f) (NH_4_)_2_SO_4_ (ultramafic soil with (NH_4_)_2_SO_4_; T6), and (g) Ca(H_2_PO_4_)_2_ (ultramafic soil with Ca(H_2_PO_4_)_2_; T7). The dose of cow manure (Florovit) used in the manure treatment reflected 0.5 g of N per 1 kg of ultramafic soil (N content in cow manure was checked using a Vario Macro Cube Analyzer from Elementar). In the rosahumus treatment, the pots were watered with 1 g of rosahumus dissolved in 1 dm^3^ of deionized water. The content of C in the rosahumus treatment was 43.5 ± 6.97% (checked using the Tiurin method). In the KNO_3_ variant, the amount of K was 0.13 g (and N content was 0.04 g N) per 1 kg of ultramafic soil. In the lime treatment, the dose of lime (Florovit, 12.5 g of lime per 1 kg of ultramafic soil) was calculated based on a short-term (24 h) lime requirement test (the amount of lime which adjusts the pH of the soil up to approximately 7). For the final two treatments, the dose of N was 0.15 g and the dose of P was 0.07 g per 1 kg of ultramafic soil in the (NH_4_)_2_SO_4_ treatment and the Ca(H_2_PO_4_)_2_ treatment, respectively. Pure KNO_3_, (NH_4_)_2_SO_4_, and Ca(H_2_PO_4_)_2_ were purchased from Agra.

All pots (except those used for the rosahumus treatment) were watered using deionized water. All pots were watered at 60% (± 15%) of capillary capacity. Before sowing, seeds of *B. napus* were dressed with Orius Extra 02 WS Adama Essentials, and after germination seedlings were sprayed with fungicide (Discus 500 WG Sumin) to prevent and control powdery mildew. Undesirable insects (i.e., *Trialeurodes vaporariorum*) were caught using a yellow sticky trap (Agrecol). The pots were watered at the beginning of the experiment, then after 5 days, ten *B. napus* seeds were sown into each pot. Five seedlings were left in each pot after germination. After 60 days since the experiment started, the leaves and roots were harvested, rinsed in fresh water and deionized water, and air dried. The leaves were weighed to calculate their yield of metallic elements, as a product of the means concentration of metallic elements in the leaves and the means of dry biomass of the leaves (Nkrumah et al., 2019a). Soil from the pots was air-dried and sieved using a 2-mm stainless-steel sieve. Fine earth (< 2 mm) was used for further analysis.

### Soil solution collection

Soil solutions were collected from the pots twice: (a) before sowing the seeds (on the fourth day after watering had begun) and (b) before harvesting the plants (on the 59th day after watering had begun). Soil solutions were collected using MicroRhizon samplers from Rhizosphere Research Products B. V. (Wageningen, Netherlands) equipped with disposable luer lock syringes. The concentration of Ca, Mg, K, Ni, Cr, and Co was measured by means of inductively coupled plasma–optical emission spectroscopy (ICP-OES) using a Perkin Elmer Avio 200 (Waltham, Massachusetts, USA; the same instrument was used for other analyses in this study). The parameters of the analysis and the instrumental conditions are presented in Appendix 1. The pH of soil solutions was measured using an Elmetron CP-505 pH meter equipped with an ERH-111 electrode (No. 9997; the same pH meter was adopted for other measurements of pH in this study).

### Physicochemical properties of soils

Physicochemical properties of soils were measured by means of methods described in Van Reeuwijk (2002) and Pansu and Gautheyrou (2006). Soil pH was measured from soil suspension with Milli-Q H_2_O and 1 M KCl (1:2.5 w/v ratio) after equilibration for 24 h. Exchangeable bases (Ca^2+^, Mg^2+^, K^+^, Na^+^) were analyzed after soil extraction, where an aliquot of the soil sample (approximately 2 g) was mixed with 50 cm^3^ of 1 M CH_3_COONH_4_ (pH 7). The suspension was left for 3 days and occasionally agitated. The content of Ca, Mg, K, and Na in filtrated suspensions was measured using ICP-OES, and presented in electrochemical units (cmol_c_/kg; Van Reeuwijk, 2002). Hydrolytic acidity (Hh; in cmol_c_/kg) was analyzed in triplicates after 40 g of soil was agitated with 0.5 M Ca(CH_3_COO)_2_ at pH 8.2. After 2 h of extraction, the filtrated suspensions were titrated using 0.1 M NaOH with several drops of phenolphthalein until a light pink color was visible. Effective cation exchange capacity (CEC) was calculated as the sum of exchangeable bases and hydrolytic acidity. Base saturation (BS) was calculated as the percentage of the sum of exchangeable bases in relation to CEC. Soil buffer capacity was analyzed using pH measurement of soil suspensions with different concentrations of HCl and NaOH (Shi et al., 2019) since no standardized methods exist (Nelson & Su, 2010; Appendix 1). The potential of the soil for acid neutralization was estimated based on the method adopted for mine wastes (Potysz et al., 2017). Details about methodology for acid neutralization potential analysis are presented in Appendix 1. The zero point of charge (pH_0_; the value of pH for which the total net charge is canceled) of the control soil was measured following Pansu and Gautheyrou (2006; Appendix 1).

### Chemical analysis of soils and plants

Aliquots of the milled soil and rock sample (100 mg) were digested using an Ethos Up microwave mineralization system (Milestone, Italy) with 5 cm^3^ HF, 4 cm^3^ H_2_SO_4_, 2 cm^3^ HNO_3_, and 1 cm^3^ HCl (Sigma Aldrich). Afterwards, the digested samples were diluted with 1.5 cm^3^ H_2_SO_4_ and Milli-Q water (18.2 MΩ·cm) up to 50 cm^3^. Certificated reference material (Metranal 34; Analytika, Czech Republic) was digested together with the soil samples (Appendix 1). Aliquot of the plant samples (500 mg) was digested with 10 cm^3^ of concentrated HNO_3_ using an Ethos Up microwave mineralization system. After the digestion, the solutions were diluted up to 50 cm^3^ with Milli-Q water. Certificated reference material 1573a, tomato leaves (National Institute of Standards and Technology, Gaithersburg, USA), was processed in the same way as the plant samples from the pot experiment. Reagent blank samples were operated in the same way as soil and plant samples. The C, N, and S content in the soil samples and plant materials was measured using a Vario Macro Cube Analyzer (Elementar). The following certificated reference materials were analyzed alongside the samples: B2186 soil standard (loamy) and B2166 birch leaf standard (from Elemental Microanalysis Elemental Lab; Appendix 1).

### Mineralogical analysis

The mineral composition of the fine earth of control soil and milled rocks was analyzed using the powder X-ray diffraction method (Bruker AXS D5005 diffractometer; Appendix 1). The clay fraction (2–0.2 µm and < 0.2 µm) of the soils was separated using Jackson’s (1969) procedure that removes carbonates, divalent cations, organic matter, and Fe and Mn oxides from soil. After the experiment, the thin polished sections were prepared from soils and were examined using a scanning electron microscope (JSM IT-100; JEOL) coupled with an energy-dispersive X-ray spectrometer (x-act, Oxford Instruments; Appendix 1).

### Statistical analysis

The chemical composition of plants between treatments was compared by means of one-way ANOVA followed by the Tukey post hoc test, as well as the Kruskal–Wallis test followed by the Conover–Iman post hoc test. These statistical tests were selected after checking for the normal distribution of results (using the Shapiro–Wilk test) and homogeneity of variance (using the Levene test). In this study, the significance level (α) was 0.05.

## Results

### Mineral composition and physicochemical and chemical properties of soils

**Fig. 1:**
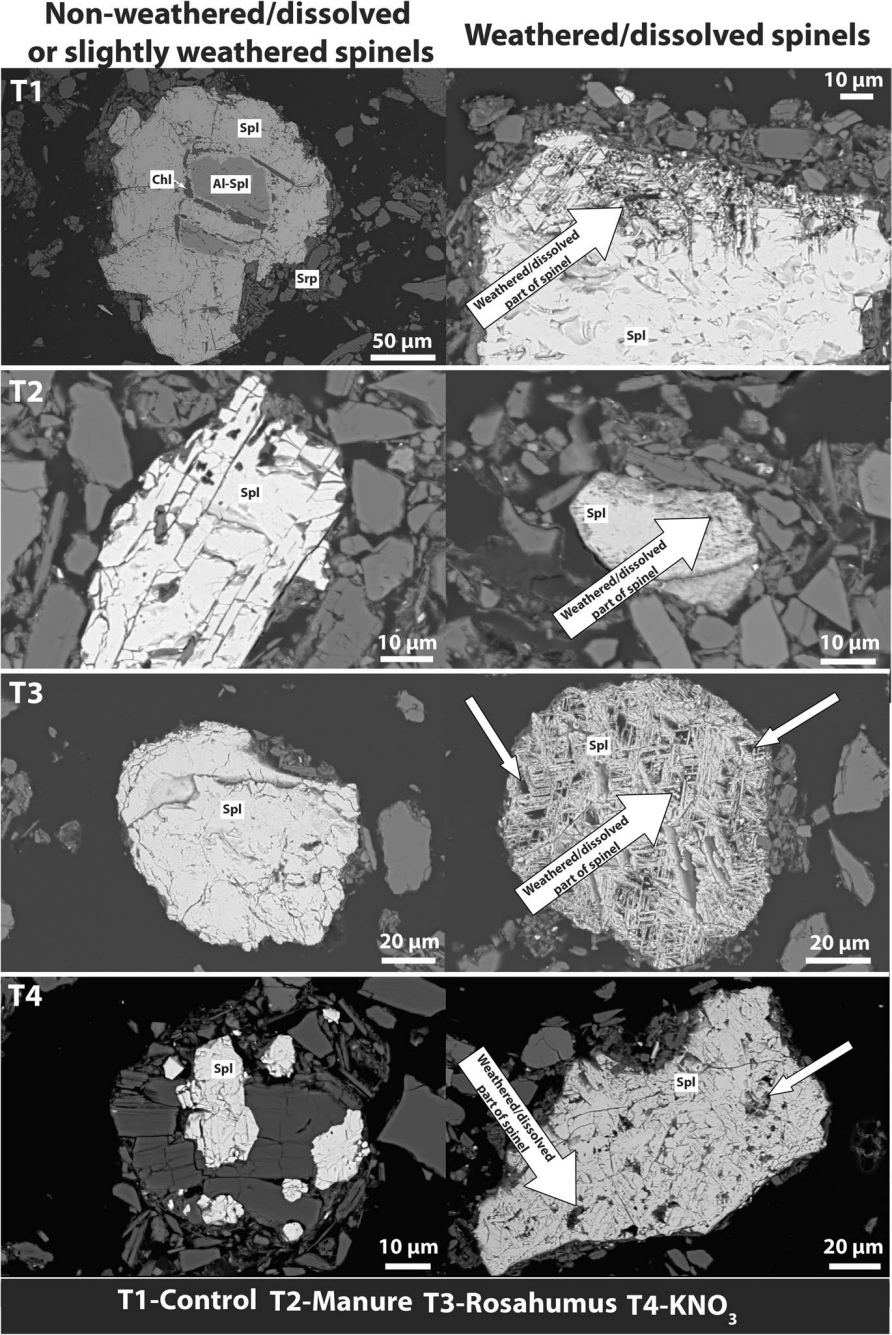
SEM images of weathering/dissolving features in spinels from control, manure, rosahumus, and KNO_3_ treatments (T1, T2, T3, AND T4, respectively; Spl—spinel, Al-Spl—Al spinel, Srp—serpentine, Chl—chlorite)

**Fig. 2:**
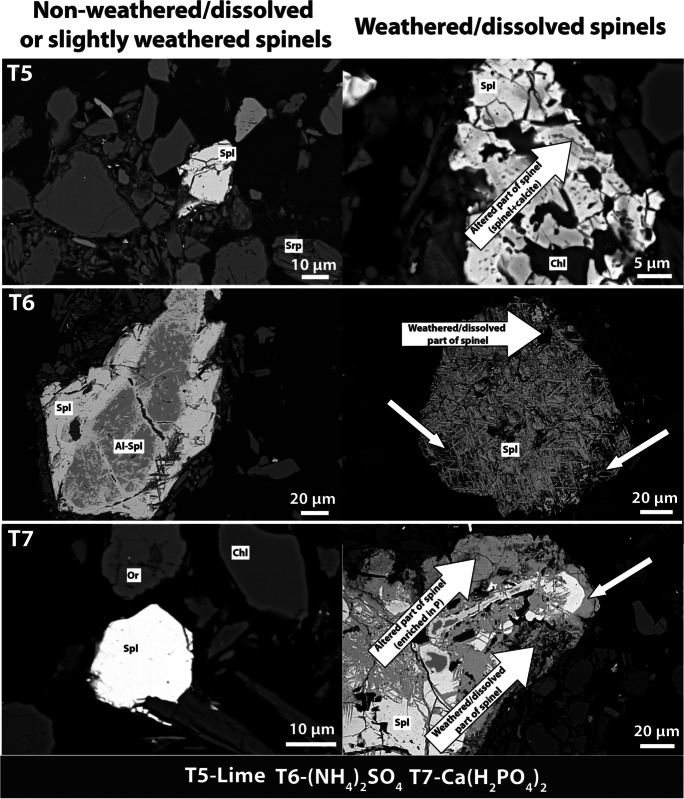
SEM images of weathering/dissolving features in spinels from lime, (NH_4_)_2_SO_4_, and Ca(H_2_PO_4_)_2_ treatments (T5, T6, and T7, respectively; Spl—spinel, Al-Spl—Al spinel, Srp—serpentine, Chl—chlorite, Or—orthoclase)

**Fig. 3:**
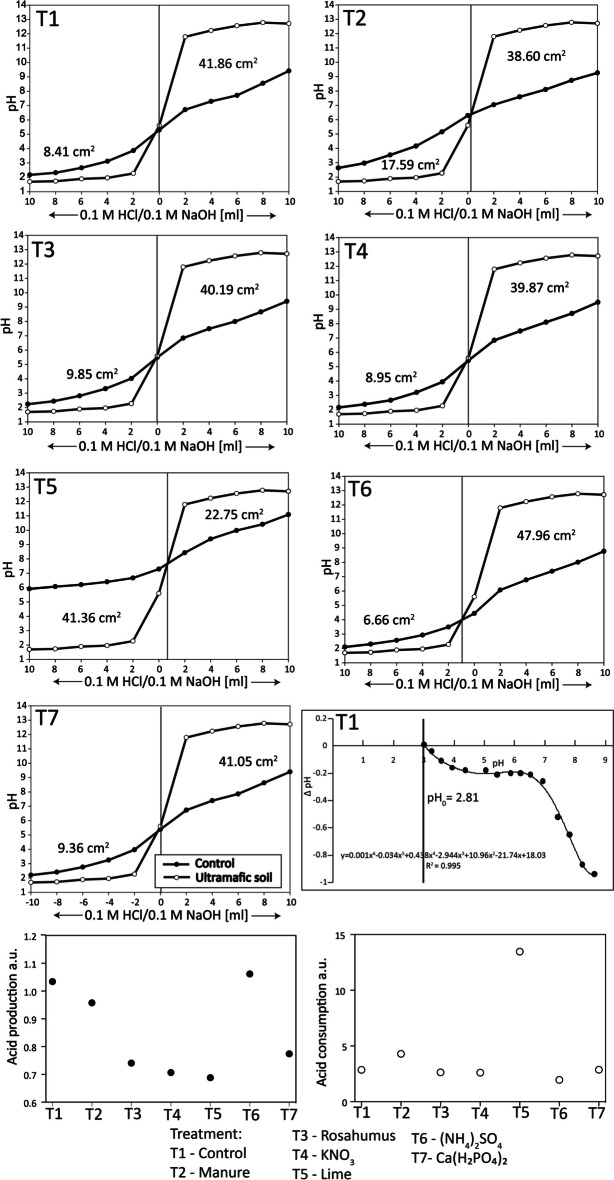
Buffer capacity of control and fertilized ultramafic soils (T1-control, T2-manure, T3-rosahumus, T4-KNO_3_, T5-lime, T6-(NH_4_)_2_SO_4_, and T7-Ca(H_2_PO_4_)_2_ treatments) and pH_0_ of control soil

Analysis of the mineral composition of rock fragments from the soil skeleton in the studied soils revealed the presence of serpentine and spinel group minerals, confirming that the soil in the arable field in Jordanów developed on serpentinite. The fine earth of the ultramafic soil in this locality is characterized by the presence of serpentine group minerals with admixtures of quartz and feldspars (Appendix 2). Furthermore, the SEM analysis revealed the presence of chlorite and allowed us to distinguish two types of spinels (Al-rich spinel and magnetite; Figs. 1 and 2). In all treatments, both not-weathered and altered spinels were observed. The alteration depended on the treatment. For example, parts of spinels enriched in Ca were observed in the lime treatment, and parts of spinels enriched in P were visible in the Ca(H_2_PO_4_)_2_ treatment. Also, spinels from the (NH_4_)_2_SO_4_ and rosahumus treatments were the most extensively altered (free spaces were noted inside the individual grains). Analysis of the clay fraction of the soils confirmed the presence of smectite, chlorite, and serpentine with admixtures of quartz and mica. The pH and pH_0_ in the control sample of ultramafic soil from Jordanów were 5.3 and 2.8, respectively (Table 1; Fig. 3). The soil additives used in this study either decreased the pH_H2O_ of the soil ((NH_4_)_2_SO_4_; pH_H2O_ 4.3) or increased it (other treatments, up to pH_H2O_ 7.5 in lime). Acidity had a close relationship with the buffer properties of soils. The lime treatment had the highest acid buffer capacity (the buffer area was 41 cm^2^), followed by manure, rosahumus, Ca(H_2_PO_4_)_2_, KNO_3_, and (NH_4_)_2_SO_4_ (Fig. 3). The highest acid buffer capacities in lime and manure were accompanied by the highest acid consumption rates. Despite that, manure’s potential for acid production is relatively high. On the other hand, ultramafic soil treated with (NH_4_)_2_SO_4_ exhibited the highest alkaline buffer capacity (buffer area was 48 cm^2^), whereas soil treated with lime exhibited the lowest (buffer area was 23 cm^2^). The CEC was within the range from 9 cmol_c_/kg (KNO_3_ treatment) up to 16 cmol_c_/kg (lime treatment). Base saturation was within the range from 43% for the (NH_4_)_2_SO_4_ treatment up to 94% for the lime treatment, whereas base saturation was 53% for the control. It should also be noted that ultramafic soil treated with manure exhibited relatively high base saturation (72%).

**Table 1:** Physicochemical properties of ultramafic soils in the pot experiment (results are presented as mean values ± SD from three independent measurements)

Treatment	pH	pH	Ca^2+^	Mg^2+^	Na^+^	K^+^	Hh	CEC	BS	Ca/Mg
	H_2_O	KCl	[cmol ( +) kg^−1^]						[%]	
T1-Control	5.29 ± 0.07	4.54 ± 0.10	2.57 ± 0.21	1.90 ± 0.09	0.01 ± 0.01	0.48 ± 0.03	4.32 ± 0.21	9.28 ± 0.14	53.4 ± 2.83	1.35
T2-Manure	6.27 ± 0.01	5.76 ± 0.03	4.59 ± 0.17	2.35 ± 0.13	0.01 ± 0.01	0.70 ± 0.08	2.92 ± 0.10	10.8 ± 0.33	72.4 ± 1.43	1.95
T3-Rosahumus	5.57 ± 0.06	4.68 ± 0.04	2.76 ± 0.10	1.94 ± 0.10	0.01 ± 0.01	0.73 ± 0.04	4.00 ± 0.05	9.43 ± 0.22	57.6 ± 0.96	1.42
T4-KNO_3_	5.46 ± 0.07	4.52 ± 0.08	2.38 ± 0.17	1.80 ± 0.17	< 0.01 ± < 0.01	0.49 ± 0.04	4.00 ± 0.15	8.68 ± 0.32	53.9 ± 1.28	1.32
T5-Lime	7.46 ± 0.06	7.14 ± 0.06	12.6 ± 0.99	1.78 ± 0.99	< 0.01 ± < 0.01	0.43 ± 0.01	0.89 ± 0.09	15.9 ± 1.01	94.4 ± 0.70	7.17
T6-(NH_4_)_2_SO_4_	4.31 ± 0.07	3.87 ± 0.05	2.20 ± 0.20	1.80 ± 0.20	< 0.01 ± < 0.01	0.25 ± 0.02	5.55 ± 0.07	9.80 ± 0.16	43.4 ± 1.62	1.22
T7-Ca(H_2_PO_4_)_2_	5.42 ± 0.02	4.54 ± 0.01	2.59 ± 0.13	1.88 ± 0.13	0.02 ± 0.01	0.45 ± 0.02	4.06 ± 0.08	8.99 ± 0.24	54.8 ± 1.01	1.38

The chemical composition of the soil samples is presented in Table 2. Soil additives mostly affected the content of macroelements, whereas the content of metallic elements was stable in all treatments. CaO content ranged from 0.39% for the control sample and the (NH_4_)_2_SO_4_ treatment up to 0.86% for the lime treatment. MgO, K_2_O, and Na_2_O contents for all treatments did not exceed 8, 1.5, and 0.5%, respectively. The highest metallic element contents were observed for Fe_2_O_3_ and Al_2_O_3_ (with Fe_2_O_3_ content of up to 4.40% in the manure treatment). Nickel, Cr, and Co contents were approximately 670, 1500, and 45 mg kg^−1^, respectively, for all treatments. In turn, manganese content did not exceed 500 mg kg^−1^ (Table 2).

**Table 2:** Chemical composition of ultramafic soils in the pot experiment and fragments of serpentinites from skeletal fraction of soils (results are presented as mean values ± SD from three independent measurements)

Treatment	CaO	MgO	K_2_O	Na_2_O	Fe_2_O_3_	Al_2_O_3_	Ni	Cr	Co	Mn	C	N	S
	[wt.%]						[mg kg^−1^]				[wt.%]		
Soils													
T1-Control	0.39 ± 0.01	7.44 ± 0.48	1.30 ± 0.01	0.48 ± 0.01	4.31 ± 0.11	3.13 ± 0.61	662 ± 4.52	1543 ± 68.5	44.8 ± 0.05	430 ± 8.54	1.67 ± 0.03	0.17 ± < 0.01	0.04 ± 0.01
T2-Manure	0.48 ± 0.02	7.44 ± 0.22	1.28 ± 0.02	0.47 ± 0.01	4.40 ± 0.14	2.93 ± 0.25	665 ± 16.6	1495 ± 172	45.3 ± 1.51	440 ± 13.5	1.98 ± 0.04	0.20 ± 0.01	0.03 ± < 0.01
T3-Rosahumus	0.40 ± 0.01	7.36 ± 0.10	1.30 ± 0.01	0.48 ± 0.01	4.33 ± 0.03	2.87 ± 0.39	664 ± 3.12	1546 ± 75.6	44.4 ± 0.53	435 ± 9.28	1.71 ± 0.02	0.18 ± < 0.01	0.02 ± < 0.01
T4-KNO_3_	0.39 ± 0.01	7.44 ± 0.31	1.28 ± 0.02	0.47 ± < 0.01	4.38 ± 0.04	3.14 ± 0.32	670 ± 9.27	1519 ± 136	44.8 ± 0.89	426 ± 3.86	1.67 ± 0.01	0.17 ± < 0.01	0.02 ± < 0.01
T5-Lime	0.86 ± 0.13	6.13 ± 1.52	1.28 ± 0.01	0.47 ± < 0.01	4.01 ± 0.25	1.96 ± 0.49	676 ± 10.7	1514 ± 85.9	44.8 ± 0.72	417 ± 26.0	1.77 ± 0.05	0.17 ± 0.01	0.02 ± < 0.01
T6-(NH_4_)_2_SO_4_	0.39 ± 0.02	7.37 ± 0.53	1.29 ± 0.03	0.48 ± 0.01	4.33 ± 0.13	2.77 ± 0.17	662 ± 17.4	1501 ± 104	44.7 ± 1.54	424 ± 22.8	1.65 ± 0.02	0.18 ± 0.01	0.03 ± < 0.01
T7-Ca(H_2_PO_4_)_2_	0.42 ± 0.02	7.68 ± 0.34	1.29 ± 0.01	0.47 ± 0.01	4.38 ± 0.12	2.91 ± 0.15	671 ± 1.24	1581 ± 74.1	45.5 ± 0.59	447 ± 22.8	1.66 ± 0.03	0.17 ± < 0.01	0.03 ± < 0.01
Rock													
Serpentinite	0.02 ± < 0.01	8.91 ± 1.52	0.01 ± < 0.01	< 0.01 ± < 0.01	6.23 ± 0.25	0.15 ± 0.05	1654 ± 29.2	3136 ± 42.6	81.6 ± 1.26	588 ± 20.4	nd	nd	nd

*nd* not determined

### Soil solution

Although chemical extractions provide fast information about the phytoavailability of metallic elements in soils, direct analysis of soil solutions collected from the rhizosphere seems to be a better way of understanding the actual interactions in a soil–root system. We collected soil solution from pots at two stages: before sowing and before harvesting and characterized the pH and chemical composition of the samples at both stages (Table 3; Appendix 3). An increase in pH between sowing and harvesting was observed in the control soil solution, and soils treated with rosahumus, KNO_3_, and Ca(H_2_PO_4_)_2_. On the other hand, a decrease in pH was observed for the manure and (NH_4_)_2_SO_4_ treatments, whereas the pH for the lime treatment was relatively stable between stages. The pH levels lower than in the control sample were noted before sowing for the KNO_3_, (NH_4_)_2_SO_4_, and Ca(H_2_PO_4_)_2_ treatments, whereas only (NH_4_)_2_SO_4_ treatment had lower than control pH before harvesting. The concentration of Ca, Mg, and K in the soil solutions markedly decreased between sowing and harvesting. For example, the concentration of Mg before sowing for the KNO_3_ treatment was 100 mg dm^3^, whereas before harvesting it was down to 3.4 mg dm^3^ (Table 3; Appendix 3). In terms of health risks Ni, Cr, and Co are of concern (Kierczak et al., 2021), and of those Ni had always the highest concentration and Co the lowest in all the soil solutions. Nickel concentration generally decreased between sowing and harvesting for all treatments except for (NH_4_)_2_SO_4_, in which the Ni concentration remained relatively constant between both stages. The highest concentration of Ni in a soil solution before harvesting was in the (NH_4_)_2_SO_4_ treatment (1960 µg dm^3^), and the lowest was in the lime treatment (244 µg dm^3^). In almost all treatments, an increase in Cr concentration was observed between sowing and harvesting (the exceptions were the manure and (NH_4_)_2_SO_4_ treatments). The most marked increase in Cr concentration was observed for the rosahumus treatment (from 13 µg dm^3^ up to 38 µg dm^3^). Finally, Co concentration decreased between sowing and harvesting for all treatments except rosahumus and (NH_4_)_2_SO_4_. As with Ni, the highest concentration of Co was noted for the (NH_4_)_2_SO_4_ treatment (up to 299 µg dm^3^).

**Table 3:** Nickel, Cr, Co, Ca, Mg, K concentration, and pH of soil solution collected using MicroRhizon samplers (results are presented as mean values ± SD from three independent measurements; median values are in italics; the same letters indicate one group according to post hoc tests)

Treatment	Ni	Cr	Co	Ca	Mg	K	pH
	[µg dm^3^]			[mg dm^3^]			
Before sowing							
T1-Control	393 ± 12.7(a,b) *387*	9.06 ± 1.70(a) *9.22*	3.27 ± 0.79(a) *3.27*	78.0 ± 14.0(a) *78.3*	64.9 ± 13.3(a) *58.5*	71.9 ± 12.8(a,b) *67.5*	4.85 ± 0.07(a,c) *4.87*
T2-Manure	659 ± 95.6(c,d) *698*	18.4 ± 3.56(b) *18.3*	38.7 ± 15.1(b,c) *39.5*	145 ± 24.6(c,d) *136*	116 ± 22.6(b) *112*	275 ± 31.8(d) *271*	6.71 ± 0.11(d) *6.77*
T3-Rosahumus	425 ± 34.9(b) *418*	12.6 ± 1.94(a,b) *12.7*	4.81 ± 2.37(a) *5.62*	59.2 ± 10.4(a) *54.4*	55.2 ± 12.0(a) *50.6*	73.2 ± 10.5(b) *72.4*	5.05 ± 0.11(c) *5.09*
T4-KNO_3_	578 ± 58.1(c) *558*	8.81 ± 2.52(a) *8.60*	4.85 ± 1.59(a) *4.37*	110 ± 9.12(b) *109*	100 ± 14.8(b) *102*	119 ± 11.5(c) *114*	4.79 ± 0.14(a,b,c) *4.77*
T5-Lime	185 ± 39.3(a) *193*	9.37 ± 4.09(a) *11.2*	3.32 ± 2.09 *3.94*	135 ± 41.0(b,c) *124*	57.0 ± 11.5(a) *52.8*	54.4 ± 7.94(a) *50.9*	7.38 ± 0.20(e) *7.28*
T6-(NH_4_)_2_SO_4_	1960 ± 734(e) *1947*	11.8 ± 1.36(a,b) *11.9*	100 ± 30.2(c) *98.5*	294 ± 111(d) *316*	253 ± 111(c) *254*	197 ± 46.7(c,d) *199*	4.44 ± 0.15(b) *4.36*
T7-Ca(H_2_PO_4_)_2_	692 ± 46.1(d) *699*	16.7 ± 0.33(b) *16.7*	14.3 ± 3.07(b) *15.5*	112 ± 7.85(b) *112*	98.1 ± 8.38(b) *97.2*	82.8 ± 8.23(b) *78.2*	4.66 ± 0.12(a,b) *4.60*
Before harvesting							
T1-Control	320 ± 20.8(a,b) *323*	28.8 ± 4.87(b) *31.2*	2.72 ± 1.01(a) *2.65*	1.87 ± 1.58(a) *2.33*	5.57 ± 1.68(a,b) *5.88*	14.3 ± 0.67(a,b) *14.2*	5.53 ± 0.05(a,b) *5.52*
T2-Manure	380 ± 143(a,b) *460*	10.9 ± 3.06(a) *12.6*	17.9 ± 7.90(c,d) *22.1*	20.7 ± 11.9(b,c,d) *27.5*	19.7 ± 11.6(c) *26.1*	46.9 ± 21.3(c) *59.1*	6.39 ± 0.14(d) *6.32*
T3-Rosahumus	450 ± 78.7(b,c) *407*	37.6 ± 13.1(b) *30.4*	6.38 ± 1.34(b,c) *6.99*	6.84 ± 2.64(c) *8.22*	10.1 ± 3.43(b,c) *11.7*	29.3 ± 12.4(b,c) *36.0*	5.68 ± 0.03(c) *5.68*
T4-KNO_3_	283 ± 17.5(a) *291*	29.6 ± 5.68(b) *28.1*	2.84 ± 1.00(a) *2.69*	0.70 ± 0.34(a) *0.46*	3.37 ± 0.98(a) *3.58*	9.95 ± 6.02(a) *7.64*	5.64 ± 0.14(b,c) *5.59*
T5-Lime	244 ± 152(a) *190*	13.4 ± 11.0(a) *11.0*	7.21 ± 6.19(b) *4.14*	51.1 ± 22.2(d,e) *61.8*	18.9 ± 5.77(c,d) *20.7*	16.4 ± 9.47(a,b) *11.9*	7.37 ± 0.04(d) *7.36*
T6-(NH_4_)_2_SO_4_	1932 ± 1044(c) *2012*	12.7 ± 5.36(a) *14.3*	299 ± 180(d) *266*	178 ± 73.7(e) *181*	158 ± 61.4(d) *152*	49.4 ± 25.5(c) *36.5*	4.15 ± 0.40(a) *3.94*
T7-Ca(H_2_PO_4_)_2_	394 ± 61.9(a,b) *369*	31.5 ± 3.62(b) *33.2*	3.71 ± 1.19(a,b) *4.09*	4.26 ± 3.98(a,b) *2.83*	7.51 ± 3.70(b) *6.35*	9.86 ± 4.58(a) *8.34*	5.57 ± 0.16(b,c) *5.62*

### Biomass of plants from pot experiment

The use of fertilizers can affect the yield potential of soils. Our experiment shows that manure mostly increased the biomass of the aboveground parts of *B. napus* compared to the control treatment (6.82 and 2.04 g DW, respectively; Appendix 4). Other fertilizers like (NH_4_)_2_SO_4_, KNO_3_, and Ca(H_2_PO_4_)_2_ increased the biomass of *B. napus* to a lesser extent. For example, the biomass of *B. napus* in Ca(H_2_PO_4_)_2_ was 2.25 g DW. On the other hand, rosahumus and lime did not particularly affect the biomass (2.01 g DW in both cases) of studied plant species relative to the control treatment.

### Chemical composition of plants from pot experiment

**Fig. 4:**
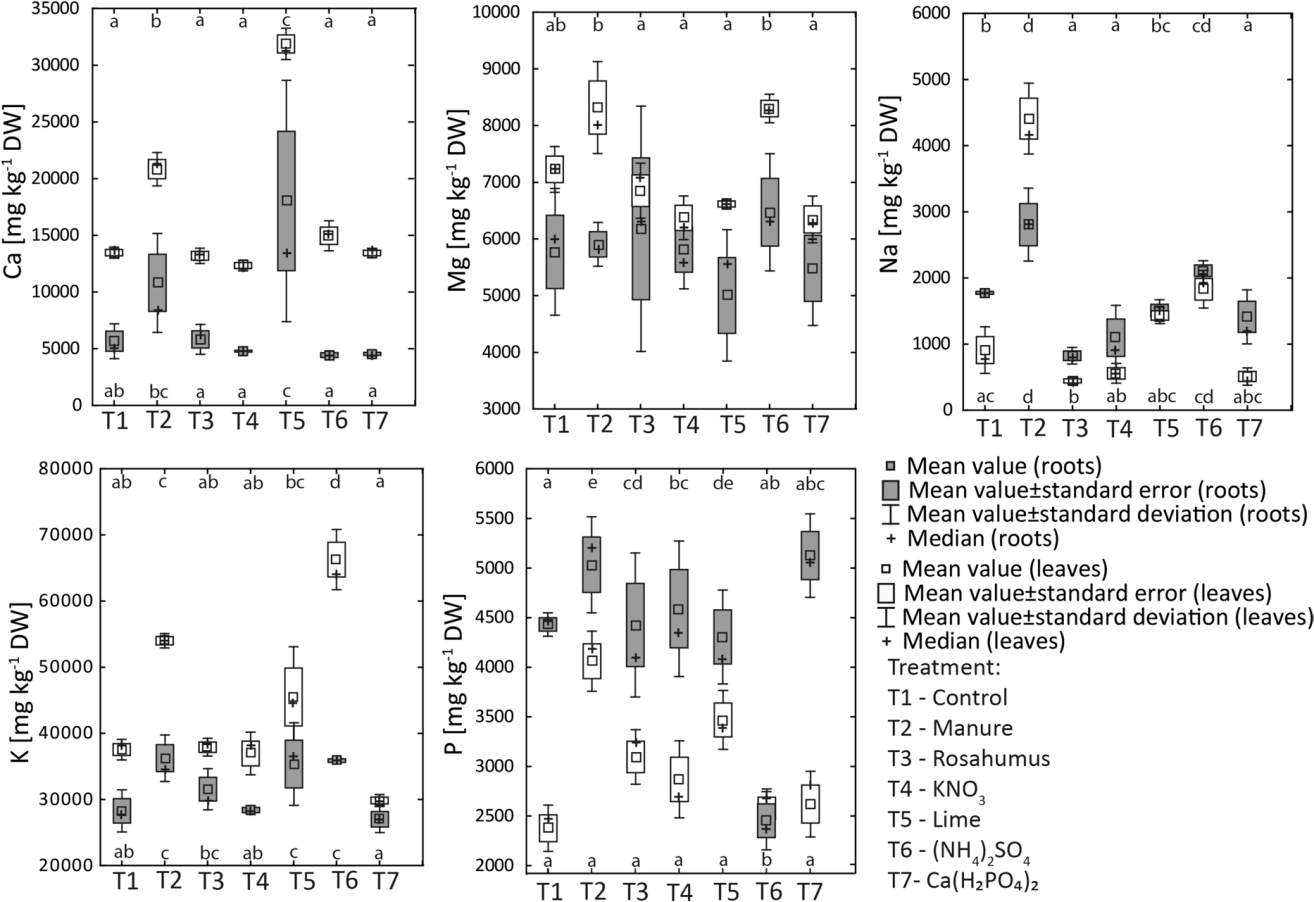
Calcium, Mg, Na, K, and P content in *Brassica napus* cultivated in control and fertilized ultramafic soils (T1-control, T2-manure, T3-rosahumus, T4-KNO_3_, T5-lime, T6-(NH_4_)_2_SO_4_, and T7-Ca(H_2_PO_4_)_2_ treatments). The results of the post hoc tests are presented in the lower (for roots) and upper (for leaves) parts of the graph. The same letters indicate one group

**Fig. 5:**
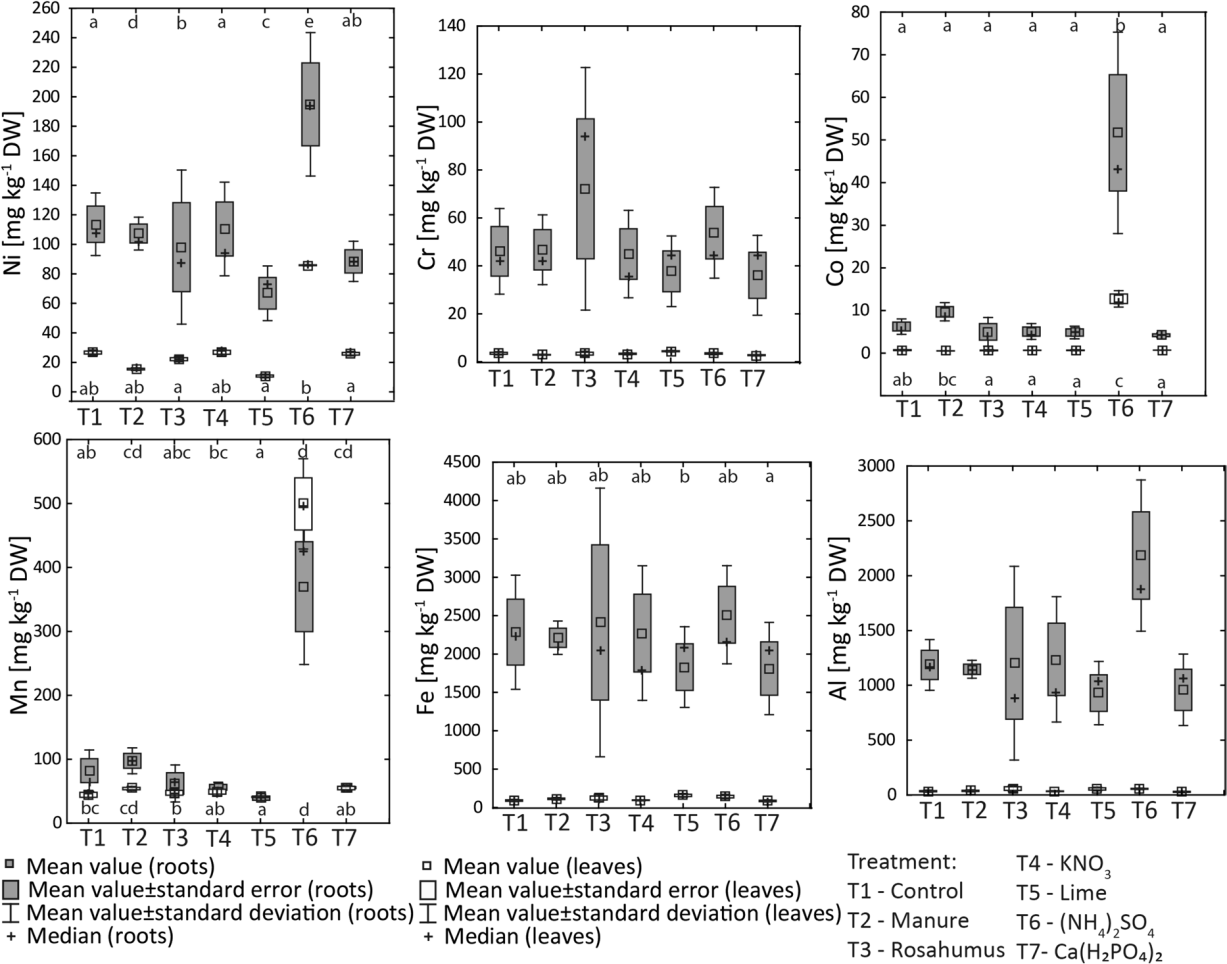
Nickel, Cr, Co, Mn, Fe, and Al content in *Brassica napus* cultivated in control and fertilized ultramafic soils (T1-control, T2-manure, T3-rosahumus, T4-KNO_3_, T5-lime, T6-(NH_4_)_2_SO_4_, and T7-Ca(H_2_PO_4_)_2_ treatments). The results of the post hoc tests are presented in the lower (for roots) and upper (for leaves) parts of the graph. The same letters indicate one group

**Fig. 6:**
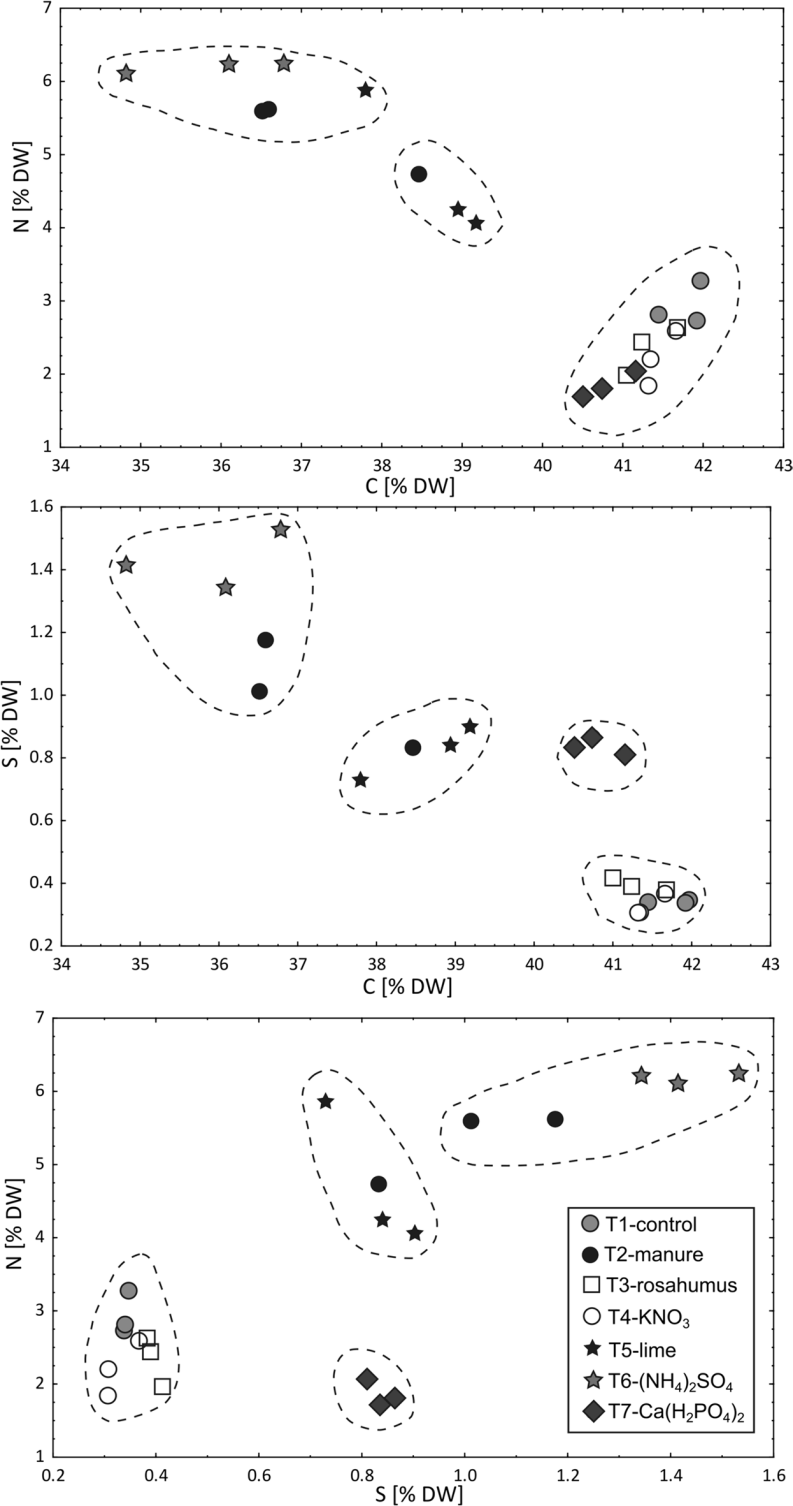
Carbon, N, and S relationships in leaves of *Brassica napus* cultivated in control and fertilized ultramafic soils (T1-control, T2-manure, T3-rosahumus, T4-KNO_3_, T5-lime, T6-(NH_4_)_2_SO_4_, and T7-Ca(H_2_PO_4_)_2_ treatments)

The chemical composition of the plants grown in the pot experiment is presented in Figs. 4, 5, and 6 and Appendix 4. It can be observed that for almost all treatments, the content of Ca, Mg, and K was higher in the leaves of *B. napus* than in the roots. In contrast, for almost all treatments, the content of Na and P was higher in the roots than in the leaves. Furthermore, for almost all treatments, the Ni, Cr, Co, Mn, Al, and Fe content in *B. napus* was higher in the roots than in the leaves. The one exception was the (NH_4_)_2_SO_4_ treatment, in which the Mn content was higher in the leaves than in the roots.

Calcium content in the roots of *B. napus* was higher for the lime and manure treatments (3.19% for lime and 2.09% for manure) relative to other treatments (i.e., in control 1.35%), whereas Mg content in roots was similar for all treatments. Among the other studied macroelements, the most visible is the increase of P, Na, and K content in roots of *B. napus* in manure and P content in Ca(H_2_PO_4_)_2_ compared to the other treatments (Fig. 4; Appendix 4).

The highest Ni in roots was observed for (NH_4_)_2_SO_4_ treatment and the lowest for lime and Ca(H_2_PO_4_)_2_ treatments (Fig. 5). Chromium content was similar in *B. napus* roots for all treatments except for rosahumus, for which roots were characterized by higher Cr content (72 mg kg^−1^ DW) relative to the other treatments. The highest Co, Mn, and Al contents in roots were noted for the (NH_4_)_2_SO_4_ treatment. Iron contents in *B. napus* roots were similar for almost all treatments.

The highest Ca content in leaves was observed for manure and lime treatments, whereas the highest Mg was noted for manure and (NH_4_)_2_SO_4_. Among the other studied macroelements, the noticeably higher contents of P and K were observed in leaves of *B. napus* in manure relative to control treatment (0.41 and 0.34% DW, respectively, for P and 5.40 and 3.75% for K, respectively).

Nickel content in *B. napus* leaves was lower for the lime (11 mg kg^−1^ DW), manure (15 mg kg^−1^ DW), and rosahumus (22 mg kg^−1^ DW) treatments relative to the control sample (27 mg kg^−1^ DW). In contrast, the (NH_4_)_2_SO_4_ treatment increased the accumulation of Ni in *B. napus* leaves (86 mg kg^−1^ DW). No remarkable effect of KNO_3_ and Ca(H_2_PO_4_)_2_ soil additives on Ni accumulation by leaves of *B. napus* was observed (27 and 26 mg kg^−1^ DW, respectively). There was no significant variation in Cr content in *B. napus* leaves for any treatment with lime treatment characterized by slightly higher values compared to other treatments. Also, the Co content in *B. napus* leaves was similar for all treatments (i.e., 0.70 mg kg^−1^ DW in control) except for the (NH_4_)_2_SO_4_ treatment (13 mg kg^−1^ DW). The highest Mn content was observed for the (NH_4_)_2_SO_4_ treatment (499 mg kg^−1^ DW) compared to the other treatments (i.e., 45 mg kg^−1^ DW in the control sample). Aluminum content in *B. napus* leaves ranged from 28 mg kg^−1^ DW for the Ca(H_2_PO_4_)_2_ treatment to 58 mg kg^−1^ DW for the rosahumus treatment. Iron content in *B. napus* leaves ranged from 86 mg kg^−1^ DW for the Ca(H_2_PO_4_)_2_ treatment up to 158 mg kg^−1^ DW for the lime treatment.

Consistent positive correlations between N and S and negative between C and both N and S were observed for *B. napus* leaves (Fig. 6). The highest content of N and S was observed in plants treated with (NH_4_)_2_SO_4_ (6.25 and 1.23% DW, respectively). Meanwhile, the highest content of C was found in the control sample (up to 41.97% DW).

## Discussion

Crop yield depends on many factors, such as the physical and chemical properties of soils, the presence of microorganisms, climate conditions, agrotechnical treatments, and the use of soil additives. Ultramafic soils are characterized by unfavorable properties for agriculture. However, many studies and field observations show that such specific soils are intended for plant cultivation (i.e., Hseu & Lai, 2017; Kierczak et al., 2021). It is therefore important to study the effect of soil additives on the properties of ultramafic soils and the phytoavailability of metallic elements.

### The effect of soil additives on pH of ultramafic soils

Ultramafic soil from cultivated areas in Jordanów is characterized by a pH of approximately 5.3. Cambisols and Leptosols derived from ultramafic rock are generally rather neutral in pH or slightly acidic (Echevarria, 2018; Kierczak et al., 2016), but the pH of such soils can be even lower than 5 (Chardot et al., 2007). The relatively low pH of the ultramafic soil used in our study is probably related to the removal of alkaline components with the crops since alkaline components like Ca^2+^ are readily taken by plants. Plants need to maintain ion balance (Weil & Brady, 2017). Therefore, the uptake of Ca^2+^ by roots leads to the release of 2H^+^ into the soil solution, hence, the pH of the studied ultramafic soil was lower than 7. Furthermore, incorrect fertilization (no liming) strategies carried out by the farmer can be responsible for relatively low pH. In turn, soil acidification was observed in (NH_4_)_2_SO_4_ treatment and it is mostly attributed to nitrification. In (NH_4_)_2_SO_4_ treatment, the NH_4_^+^ reacts with oxygen in soil air leading to the production of H^+^, NO^2−^, and H_2_O. The addition of S-rich soil additives to soils in different forms can also result in soil acidification (Nkrumah et al., 2016). For example, the reaction of elemental S in soils leads to the production of H^+^, SO_4_^2−^, and CH_2_O. Nevertheless, most of the soil additives used in our experiment increase the pH of soils (except for (NH_4_)_2_SO_4_). The increase in soil pH, compared to control, was most visible after the addition of lime and manure. The effect of lime on the rise in pH results from the dissolution of CaCO_3_ in soils under the influence of H_2_O and CO_2_. The soluble Ca-bicarbonates are formed and Ca^2+^ replaces the exchangeable position in a colloidal complex of soil (Weil & Brady, 2017). In turn, the increase of pH in manure amended ultramafic soil can be attributed to the relatively high content of bicarbonates and organic acids with carboxyl and phenolic hydroxyl groups (Whalen et al., 2000).

### The effect of soil additives on buffer properties of ultramafic soils

Besides pH, another important factor in ultramafic soils is buffer capacity. Particular attention should be paid to the buffer properties in the acid range since metallic elements can be mobilized in acid conditions. As with the pH observations, the highest acid buffer capacities and acid neutralizing potential were found for lime and manure among all studied treatments. Other soil additives have relatively low acid buffer properties, and only slight differences were observed between them. The buffering properties of lime in acid conditions are related to the dissolution of carbonates and the consumption of protons (Blume et al., 2016). The increase in soil resistance to acidification after adding manure results from the protonation of organic anions from the dissociation of weakly acidic functional groups on soil organic matter to form neutral molecules (Shi et al., 2019) Furthermore, the buffering behavior of manure-amended soil may be due to the relatively high cation exchange capacity that consequently causes more acidity to be neutralized (García-Gil et al., 2004). Manure was also the source of basic cations (Ca, Mg, and K), hence higher resistance to acidification compared to untreated soil is expected. Organic matter itself may also be responsible for increasing buffering capacity mainly due to carboxylic acid groups exhibiting pH-dependent charge (Stevenson, 1994). Rosahumus is also characterized by buffering properties in acidic conditions. According to Pertusatti and Prado (2007), commercial humic acids behave as buffers in both acidic and alkaline conditions (pH 5.5–8), but the maximum buffer capacity is at pH 6. In our study, rosahumus only exhibited buffer properties in acidic conditions; this may be related to the origin of these specific humic acids (in this case, from leonardites). The buffer properties of humic acids are related to the chemisorption of protons and hydroxyl ions (Pertusatti & Prado, 2007) and are also linked to the charge of carboxylic acid groups (Stevenson, 1994). In humic acids, many weak acid groups are dispersed on humic macromolecules. In chemisorption, the OH^−^ sticks to the surface by forming chemical bonds with weak acid groups of humic acids (Pertusatti & Prado, 2007).

Considering all studied soil properties, we note that among studied soil additives, lime and manure can be recommended for fertilization of ultramafic soils because they increase soil pH, exhibit acid buffering properties, and are sources of exchangeable Ca. On the other hand, (NH_4_)_2_SO_4_ must not be used for ultramafic soils, as this soil additive further decreases the pH of soils creating unfavorable conditions.

### The effect of soil additives on Ni, Cr, and Co mobilization from ultramafic soils to soil solutions

Analysis of soil solutions from the pot experiment demonstrates that the release of metallic elements from ultramafic soils to soil solutions can be considered a serious environmental hazard. Furthermore, the use of fertilizers for ultramafic soils and root exudation can change metallic elements concentrations in soil solution. Therefore, chemical composition of soil solution during plants growth (i.e., before harvesting the plants) plays an important role in understanding metallic elements mobilization in agricultural ultramafic soils. Our study demonstrated that (NH_4_)_2_SO_4_ substantially increases Ni and Co content in soil solution relative to the control. Such observation results from the ability of (NH_4_)_2_SO_4_ to lower the pH of soils and soil solution. Furthermore, mobilization of Ni, Cr, and Co from ultramafic soils to soil solutions was also observed in rosahumus and Ca(H_2_PO_4_)_2_ treatments relative to control. Similarly, Chaab et al. (2016) provided evidence for an increased Cr mobility in soils following the addition of humic acids. The increase of Cr in soil solutions from pots filled with ultramafic soil treated with rosahumus may be the result of the interaction between Cr, humic acids, and Fe (Zhilin et al., 2004). It was suggested that the presence of Fe^3+^ (as expected in our pot experiment) and humic acids obtained from leonardites does not reduce Cr^6+^ to Cr^3+^ (more and less mobile form of Cr, respectively). Furthermore, the reduction of Cr^6+^ undergoes mostly at pH 5.4, but in alkaline conditions does not (independently of the origin of humic acids). Thus, such mechanisms can undergo in our experiment since rosahumus in our studies was isolated from leonardites and a slight increase of pH is visible in soil and soil solution after the rosahumus addition (pH 5.68) compared to the control ultramafic soil (pH 5.53). In contrast, Kim et al. (2005) observed in a laboratory experiment that an increase in humic acid concentration results in an increase in the pH of the solution and in sorption of Co. This observation was explained by the deprotonation of functional groups in humic acids under higher pH conditions. However, soil (in the general sense) is variable and is composed of many elements that interact with each other. The interactions between metallic elements, humic acids, and clay minerals are very important for the mobilization of metallic elements in soils because of the sorption properties of clay minerals. In our study, the clay fraction of ultramafic soil was characterized by the presence of smectite. Hu et al. (2012) demonstrated that the sorption of humic acids on Ca-montmorillonite (smectite) decreases with the increase of pH (at pH 8, only half of the humic acid content is adsorbed on montmorillonite). Therefore, residual humic acids still exist in the solution and form soluble Ni–humic acid complexes, which decrease Ni sorption on montmorillonite (Hu et al., 2012). This mechanism may explain, to some extent, the increase of Ni, Cr, and Co content in soil solution treated with rosahumus compared to the control sample since a slight increase in the pH of the soil solution (and the soil) was noted for the rosahumus treatment compared to the control ultramafic soil. However, other studies have shown that the selected metallic elements' adsorption on montmorillonite does not correlate with the amount of humic acid adsorbed on montmorillonite (Liu & Gonzalez, 1999). Furthermore, our observation for Ca(H_2_PO_4_)_2_ has its confirmation in other studies. For example, Becquer et al. (2003) demonstrated that P-fertilizers increase in Cr content (up to 700 µg dm^3^) in soil solutions compared to non-fertilized ultramafic soils. Chromium (Cr^6+^) has an anionic nature. Therefore, Cr^6+^ has a high affinity to Fe-oxides, and Cr^6+^ forms an innersphere complex on Fe-oxides. After the addition of P-rich fertilizers to soils, phosphate replaces Cr^6+^ from Fe-oxides because of higher retention strength of phosphate relative to Cr^6+^ (Becquer et al., 2003). Therefore, the addition of P-rich fertilizers can desorb Cr from Fe-oxides and the increase of Cr in soil solution is noted. Presented findings demonstrate that agricultural ultramafic soils should not be fertilized with (NH_4_)_2_SO_4_, rosahumus as well as Ca(H_2_PO_4_)_2_. On the other hand, our study showed that liming is the effective process in decreasing the mobilization of Ni and Co from ultramafic soil to soil solution due to the increase of pH. Some observations were also noted between other treatments and between two stages of soil solution collection. Many factors (and interactions between them) are responsible for such observations, i.e., clay minerals, soil microorganisms, root exudates, etc. Despite the diverse effect of different soil additives on the concentration of Ni, Cr, and Co in soil solutions observed in our study, it should be stated that the release and further transport of metallic elements in soils is possible. This has far-reaching implications since soil solutions are transferred to groundwater and aquifers.

### The effect of soil additives on the phytoavailability of metallic elements

Fertilization strategy changes the uptake of macroelements and metallic elements by plants. In our study, almost all evaluated metallic elements were mostly accumulated in the roots of *B. napus* compared to the leaves. This points to an exclusion mechanism of metallic elements that has been observed in plants occupying semi-natural grasslands in ultramafic complexes in Poland (Pędziwiatr et al., 2018).

#### Phytoavailability of Ni

The Ni content was lower in roots for the lime, rosahumus, and Ca_2_(HPO_4_)_2_ treatments relative to the control sample. In leaves, a decrease of Ni accumulation was observed for the lime, manure, and rosahumus treatments compared to the control treatment. Such mechanisms of phytoavailability of Ni for *B. napus* can be the result of changes in pH and buffer properties after soil fertilization. In this context pH_0_, the state in which the total net charge of soils is zero, is important (Pansu & Gautheyrou, 2006). This parameter is important due to the sorption of ions. The pH_0_ of the control soil in this study was 2.81. In view of metallic elements' mobility in soils, it is important that with the rise of soil pH above pH_0_ the charge becomes negative. The soil solutions treated with lime and manure had the highest pH values. Furthermore, the CEC of soils in both cases was the highest relative to other treatments. Therefore, the significant increase of pH above pH_0_ was responsible for decreasing the phytoavailability of Ni (in the leaves of *B. napus*). The addition of both soil additives probably caused the highest increase of negative charge in soil compared to other treatments. Therefore, Ni cations were sorbed by soils leading to the decrease of Ni cations in soil solutions. The ability of lime to reduce Ni mobility in ultramafic soils is confirmed in this study by the lower concentrations of Ni in soil solution in lime treatment compared to control ultramafic soil (before harvesting). At the same time, the pH of soil solution in lime treatment was higher compared to control ultramafic soil. For manure, rosahumus, and Ca_2_(HPO_4_)_2_ treatments, the Ni concentration (before harvesting) in soil solutions was higher compared to soil solution from control ultramafic soils, even if the pH in these three cases was higher relative to control soil solution. It is difficult to explain such observation in relation to the accumulation of metallic elements by plants. The reason for this may be the plant’s ability to adapt to accumulation of metallic elements in various conditions, regardless the concentration of metallic elements in soil solution. Furthermore, the plants may secrete various compounds affecting accumulation of metallic elements depending on conditions. It should be also emphasized that chemical composition of soil solution can change dynamically, i.e., due to organic matter mineralization and humification in manure treatment. Development of microorganisms after the addition of organic matter to soil can also affect metallic elements phytoavailability (Fernández et al., 1999). Increase in the activity of microorganisms can reduce the phytoavailability of metallic elements. Furthermore, soil and soil solution in rhizosphere can be acidified by root exudates. Therefore, buffer properties of soils are important in relation to the change of pH and phytoavailability of metallic elements consequently. Relatively low phytoavailability of Ni in lime, manure, and rosahumus treatments in our study can be explained by the highest buffer properties of the soil with these additives under acidic conditions among the studied treatments. Therefore, these fertilizers counteract sudden changes in pH and thus the phytoavailability of Ni is relatively low. Furthermore, crystallization of secondary minerals in soils can affect the phytoavailability of Ni in this study. For example, the slight decrease of Ni content (in roots) in the Ca(H_2_PO_4_)_2_ treatment relative to the control ultramafic soil can be attributed to Ni binding in newly formed Ni phosphates (Kukier et al., 2004). Therefore, crystallization of Ni phosphates can decrease the phytoavailable pool of Ni. Nevertheless, Nkrumah et al. (2021) have mentioned that where Ni-phosphates are present in soils, Ni accumulation depends on the plant species.

Our observations in relation to the effect of lime on accumulation of Ni by *B. napus* are confirmed in other studies with laboratory and field experiments. For example, Chaney et al. (2007) observed that the sorption of Ni in soils increases when the soil is characterized by a relatively high pH and the presence of Fe oxides. This results in lower Ni uptake by plants. The decreased uptake of Ni by plants growing in soils treated with lime has also been observed in studies with *Berkheya coddii* (Robinson et al., 1999). In other studies, mimicking ultramafic soil solution, the mitigation effect of Ca on Ni toxicity for *Festuca rubra* was also confirmed and the decrease of the metallic element content in the plant was noted (Johnston & Proctor, 1981). Another experiment with plants and simulated soil solution revealed a decrease of Ni content in *Alyssum* sp. when a high dose of Ca was added to the solution (Chaney et al., 2008). Relatively low doses of Ca in solution increased Ni content in *Alyssum* sp. Similarly, Pędziwiatr et al. (2020) demonstrated that the application of Ca and Mg to Ni-enriched solution mitigates Ni toxicity during seed germination. However, Fernández et al. (1999) did not notice any effect of lime on the accumulation of metallic elements by *Agrostis stolonifera* and *Festuca rubra*.

Reduction of the translocation of metallic elements from the roots to the leaves of plants following the use of manure as a soil additive has also been observed by other authors. For example, the reduced uptake of metallic elements by *Agrostis stolonifera* and *Festuca rubra* after the addition of organic matter to ultramafic soils has been evidenced in Northwestern Spain (Fernández et al., 1999). Similarly, Naveed et al. (2021) have noted that the addition of manure to soils caused a lower uptake of Ni by *Brassica napus* growing in contaminated soils. The decreased uptake of Ni by hyperaccumulators (*Alyssum*) and non-hyperaccumulators (*Zea mays*) was also observed after the addition of manure to serpentinite mining waste and contaminated soils (Ghasemi et al., 2018a, 2018b; Rizwan et al., 2016). However, it should be stressed that despite the lower content of Ni in the leaves of *B. napus* in the manure treatment in our study, the Ni yield was slightly higher than in the control treatment (Appendix 4). Similar observations have been noted in studies using *Alyssum* sp., *Noccaea geosigense*, and *Odontarrhena* sp. (Álvarez-López et al., 2016; Ghasemi et al., 2018a, 2018b). Furthermore, Hipfinger et al. (2021) have shown that Ni yield in plants depends on the origin of the manure used to fertilize them. Cow manure was found to result in the highest Ni yield, followed by pig manure, and then by NPK fertilizer.

The role of P during Ni accumulation in our study was not significant; the Ni content of the leaves of *B. napus* was quite similar in the control and Ca(H_2_PO_4_)_2_ treatments (a slight difference was noted in the roots only). Similarly, P fertilizer was not found to affect the phytoextraction of Ni in *Alyssum murale* and *Alyssum corsicum* growing in Ni-contaminated soils (Li et al., 2003). It has also been noted in experiments with *Phyllanthus rufuschaneyi* and *Rinorea bengalensis* that there was no significant effect of the addition of P to ultramafic soils on the accumulation of Ni (Nkrumah et al., 2021). Nevertheless, Ni hyperaccumulators from Sabah have exhibited a relatively high ability to store P compared to non-hyperaccumulators growing in the same type of soils.

On the other hand, an increase in Ni uptake was apparent in the (NH_4_)_2_SO_4_ treatment compared to the control sample and other treatments (both in roots and leaves). The explanation for this is the effect of (NH_4_)_2_SO_4_ on chemical composition of soil solution. The analysis of soil solution in (NH_4_)_2_SO_4_ treatment clearly showed that (NH_4_)_2_SO_4_ addition to soils increases the Ni concentration in soil solution compared to soil solution from control ultramafic soil (approximately sixfold increase). Mobilization of Ni from ultramafic soil with (NH_4_)_2_SO_4_ to soil solution resulted from decreasing the pH in the nitrification process. Therefore, in (NH_4_)_2_SO_4_ treatment, increased pool of potentially available Ni for uptake by plants was noted. The effect of ultramafic soil pH on the accumulation of metallic elements by plants is also dependent on plant species and metallic element accumulation strategy. The acidification of ultramafic soils has been found to cause an increase in Ni yield in *Rinorea bengalensis* (despite the reduction of growth), whereas Ni uptake by *Phyllanthus rufuschanei* was lower (Nkrumah et al., 2019a, 2019b, 2019c). Furthermore, (NH_4_)_2_SO_4_ is the source of S, and the addition of S to soils increases the phytoavailability of metallic elements (Nkrumah et al., 2016; Robinson et al., 1999). Sulfur is associated with Ni; therefore, a relatively high Ni content can be stored in the vacuoles of Ni-hyperaccumulators. In our study, the role of S in interactions with Ni was also important. *B. napus* grown in soil treated with (NH_4_)_2_SO_4_ was characterized by the highest content of both Ni and S among all treatments.

#### Phytoavailability of Cr

Chromium content in roots slightly decreased in the lime and Ca(H_2_PO_4_)_2_ treatments relative to the control treatment. The mechanism of lime and Ca(H_2_PO_4_)_2_ in decreasing phytoavailability of Cr is similar to Ni (described in the previous section). In leaves, Cr content was similar for all treatments. Chromium content was higher in *B. napus* roots grown in soil treated with rosahumus and (NH_4_)_2_SO_4_ compared to the other treatments. Nevertheless, Cr concentration was higher in soil solution in rosahumus treatment compared to soil solution from control ultramafic soil, whereas such observation was not noted in (NH_4_)_2_SO_4_ treatment (before harvesting). Therefore, this suggests that humic substances can mobilize Cr that can be potentially available for plants uptake. The enhanced accumulation of Cr by the roots of *B. napus* can be explained due to the interactions between Cr, Fe, and humic acids in soils (Zhilin et al., 2004). It is possible that more mobile form of Cr (Cr^6+^) was not reduced to relatively stable form (Cr^3+^) in the case of presence of Fe^2+^ and humic acids from leonardites in soil. Furthermore, the mobilization of Cr in Polish ultramafic soils (and the accumulation of Cr by plants) is possible because Mn oxides contribute to this process (Garnier et al., 2013; Kierczak et al., 2016). In turn, enhanced accumulation of Cr by roots of *B. napus* in (NH_4_)_2_SO_4_ treatment can result from the decrease of pH, even though lower concentration of Cr was observed in soil solution in (NH_4_)_2_SO_4_ treatment relative to soil solution from control ultramafic soil. Variability in metallic elements accumulation can result from the biology of the studied species itself and may vary between species. Moreover, the SEM observations in our study reveal that spinels (Cr-carriers) can be dissolved with humic acids and that Cr is accumulated by *B. napus*. Garnier et al. (2008) observed chromite weathering leading to the liberation of Cr^3+^ to soils. This shows that spinels, which are considered resistant to weathering, can undergo weathering/dissolution in ultramafic soils. Thus, we suggest that fertilization (as shown by our pot experiment) contributes to the dissolution of spinels (and probably to the dissolution of other Cr-carriers like chlorite). Nevertheless, in the control treatment, weathered/dissolved spinels were also noted, suggesting that plowing and previous fertilization by farmers accelerates the weathering/dissolution of spinels and possibly other Cr-carriers.

#### Phytoavailability of Co, Mn, Al, and Fe

Cobalt content was similar in *B. napus* roots for all treatments except (NH_4_)_2_SO_4_ and manure treatments, for which an increase in Co content was noted compared to the other treatments. In leaves, Co content was similar for all treatments except the (NH_4_)_2_SO_4_ treatment, for which a significant increase of Co content was noted. In the (NH_4_)_2_SO_4_ treatment, relatively high phytoavailability can be explained by the low pH of the soil treated with this fertilizer (the same mechanism as for Ni). Furthermore, it is known that Co is present in a soluble form in soils with relatively low pH and is not sorbed, i.e., by Fe, Mn-oxides (Kabata-Pendias, 2011). Our results support this finding since Co concentration in soil solution in (NH_4_)_2_SO_4_ treatment is higher compared to soil solution from control ultramafic soil. In turn, manure's effect on Co's phytoavailability is opposite to that observed for Ni. The phytoavailability of Co after fertilization with organic matter can be dependent on the type of organic matter used. However, more research about the influence of manure on Co phytoavailability is needed.

A decrease of the Mn content in *B. napus* roots and leaves was mostly observed in the lime treatment compared to the control treatment. The explanation for the effect of lime on Mn uptake by *B. napus* is the same as for Ni (as discussed above). Manganese was mostly accumulated in roots and shoots in the (NH_4_)_2_SO_4_ treatment relative to the other treatments due to the ability of (NH_4_)_2_SO_4_ to acidification of soils. Furthermore, Mn was the only case where the content of a metallic element was higher in the leaves than in the roots, indicating enhanced translocation. At the same time, chlorosis occurred at the edges of leaves (Appendix 4). Chlorosis in oat leaves growing in pot experiments with ultramafic soils from Poland has been attributed to Fe deficiency (Żołnierz, 2004). However, in our study, the deficiency of Fe in plants grown in soil treated with (NH_4_)_2_SO_4_ was not a substantial problem because in this case the plants were characterized by similar or higher amounts of Fe compared to other treatments. Furthermore, the presence of S in the (NH_4_)_2_SO_4_ treatment mitigated the Fe deficiency which has been evidenced in *B. napus* (Muneer et al., 2014). Although the leaves of *B. napus* are characterized by relatively high Ni and Co contents, it seems possible that Mn translocation is the major reason for chlorosis in leaves. Marginal chlorosis caused by an excess of Mn has been observed in lettuce in other studies (Vlamis & Williams, 1973). Furthermore, the high content of Mn in the leaves of *B. napus* in our study was accompanied by a relatively low content of P in both roots and leaves. Jiang et al. (2015) provide evidence that P deficiency in soils results in intensified accumulation of Mn in *Lupinus albus*.

Lower contents of both Al and Fe were observed in the roots of *B. napus* in the lime and Ca(H_2_PO_4_)_2_ treatments compared with the other treatments. On the other hand, (NH_4_)_2_SO_4_ treatment increased the Al content in roots. The Al content in *B. napus* leaves was similar for all treatments. A slight increase of Fe content in *B. napus* leaves was observed for the lime treatment compared to the Ca(H_2_PO_4_)_2_ treatment; however, it remained similar to the Fe content in the control treatment. Thus, Ca-rich soil additives decrease the phytoavailability of Al and Fe due to the increase of soil pH, whereas the increased phytoavailability of Al in the (NH_4_)_2_SO_4_ treatment is attributable to relatively low soil pH.

### The effect of soil additives on the productivity of ultramafic soils in which *Brassica napus* is grown

The use of soil additives affects the yield potential of soils. Our experiment shows that manure mostly increased the biomass of the aboveground parts of *B. napus* compared to the control treatment. Other soil additives like (NH_4_)_2_SO_4_, KNO_3_, and Ca(H_2_PO_4_)_2_ increased the biomass of *B. napus* to a lesser extent. On the other hand, rosahumus and lime did not affect the biomass of the studied plant species or produced a lower biomass than the control treatment. The increase in *B. napus* biomass in manure treatment can be explained by the fact that in manure-amended soil increased microorganism activity could have occurred and consequently positively affected the permeability of cell membranes and thus nutrient uptake (Valdrighi et al., 1996). A similar role of manure in increasing the biomass of plants has been noted in studies of *Alyssum murale* in Albania as well as in studies of *Odontarrhena* spp. (Bani & Echevarria, 2019; Ghasemi et al., 2018a, 2018b). The dose and form of N also play a significant role in the effect of fertilizer on plant biomass. A relatively high dose of N (360 mg kg^−1^, as a solution) caused a reduction in the growth of *Phyllanthus rufuschaneyi* as well as *Rinorea bengalensis*, whereas 180 mg kg^−1^ of N did not have such an effect (Nkrumah et al., 2019a, 2019b, 2019c). The lower dose of N resulted in an increase in the height of plants, the number of leaves, and the thickness of stems (in *P. rufuschaneyi*). In our study, the addition of manure (500 mg kg^−1^ of N) to soils stimulated the growth of *B. napus*. Therefore, the addition of N in the form of manure at the beginning of plant growth seems more effective than the application of N as a solution. An increase in the biomass of *B. napus* leaves with (NH_4_)_2_SO_4_ and KNO_3_ was also observed, but this was not as high as it was with manure treatment. A positive impact of inorganic fertilizers with N on the increase of biomass of plants (i.e., *A. murale*) has been evidenced (Kanso et al., 2018). However, relatively high doses of inorganic fertilizer (i.e., (NH_4_)_2_SO_4_) must not be used due to soil salinization, which can negatively affect plant growth. A positive impact of Ca(H_2_PO_4_)_2_ on the biomass of *B. napus* was observed, although this effect was not spectacular. In other studies, the addition of P did not affect the biomass of *A. murale* (Shallari et al., 2001). In addition, other studies have demonstrated that the addition of P fertilizer to previously unfertilized ultramafic soils positively affects the biomass of plants; however, the addition of P fertilizer does not affect the biomass of plants growing in previously fertilized ultramafic soils (Nkrumah et al., 2021). This was explained by the fact that relatively high amounts of P may reduce the symbiotic development of roots and fungi. The source of P is very important since in our study the roots of *B. napus* contained similar amounts of P in the manure as well as the Ca_2_(HPO_4_)_2_ treatment, whereas a higher uptake of P in *B. napus* leaves was observed for the manure treatment than for the Ca_2_(HPO_4_)_2_ treatment (Fig. 4). It is of high importance that the addition of P to soil generally causes this element to be easily bound in the soil, inhibiting its solubility. In our case, the difference in P uptake by leaves of *B. napus* between the manure and Ca_2_(HPO_4_)_2_ treatments can be explained by the possibility to form struvite (NH_4_MgPO_4_·6H_2_O) after the fertilization of ultramafic soils with manure (Baugé et al., 2013). Struvite is considered to be more soluble, in terms of P, than Ca-phosphates. In addition, P availability from struvite is also crop specific because the root exudation of different crop species varies and consequently different forms of rhizosphere acidification can take place (Hertzberger et al., 2020). On the other hand, our study shows that the liming of ultramafic soils does not substantially affect the biomass of *B. napus*. That is consistent with studies of *Phyllanthus rufuschaneyi* and *Rinorea bengalensis* (Nkrumah et al., 2019a, 2019b, 2019c). Independently of the impact of Ca on plant biomass, this element is necessary for ultramafic soils since the content of Ca in ultramafic rocks is relatively low and a certain amount of Ca is removed with the crop each year (Nkrumah et al., 2016). Furthermore, Ca can mitigate the toxicity of Ni during seed germination (Pędziwiatr et al., 2020).

## Conclusions

Ultramafic soils are characterized by unfavorable properties for plant growth. Due to the increasing demand for food, extra land is being sought for cultivation. As a result, ultramafic soils are being incorporated into food production in countries such as Poland. However, the cultivation of such soils requires special attention. Our study aimed to determine which soil additive most effectively decreases the phytoavailability of Ni, Cr, Co, Mn, Al, and Fe while increasing the biomass of plants and the availability of beneficial elements (P and N) at the same time. In this study, we used a pot experiment to evaluate the effect of several soil additives (manure, humic acids, KNO_3_, lime, (NH_4_)_2_SO_4_, and Ca(H_2_PO_4_)_2_) on the phytoavailability of metallic elements for *B. napus* grown in serpentinite-derived ultramafic soil collected from an arable field. This study demonstrates that manure can be used in the fertilization of ultramafic soils because this soil additive improves several properties of ultramafic soils and positively affects the plant growth at the same time. Manure significantly increases the pH of soil compared to control ultramafic soil (pH 6.27 and 5.29, respectively). Furthermore, manure is characterized by relatively high buffer properties with regard to potential soil acidification (buffer area in manure-treated ultramafic soil is 17.59 cm^2^, whereas in control ultramafic soil 8.41 cm^2^). The application of manure increases the biomass of *B. napus* compared to untreated ultramafic soil (3.3-fold increase of biomass) due to the fact that manure is the source of N that promotes plants growth. Another advantage of the use of manure is the reduced uptake of Ni by *B. napus* relative to this plant from control ultramafic soil (15.43 mg kg^−1^ of Ni and 26.66 mg kg^−1^, respectively, in leaves) and enhanced uptake of P by leaves (0.41% of P and 0.24% of P in *B. napus* from ultramafic soil treated with manure and control ultramafic soil, respectively). What is more, the use of manure to fertilize of ultramafic soils is also part of the circular economy. Other soil additives, like lime, decrease the uptake of Ni. However, Ca-rich soil additives do not affect the biomass of plants grown in arable ultramafic soil. Therefore, this study provides implications to the management of cultivated ultramafic soils by farmers. Manure is recommended for the fertilization of arable ultramafic soils. The use of manure as fertilizer of ultramafic soils will enable to increase plant yield. Furthermore, the obtained crop will contain less metallic elements (at least Ni) than plants grown in unfertilized soil. On the other hand, fertilizers such as (NH_4_)_2_SO_4_ and humic acids must not be used to fertilize ultramafic soil as they increase the phytoavailability of Ni, Co, and Mn for *B. napus* (in the case of (NH_4_)_2_SO_4_) and the phytoavailability of Cr (in the case of humic acids). Undoubtedly, our study showed that soil is a dynamic system where many factors influence each other and interactions between soil, soil solution, and plants are complex. Therefore, the use of soil additives in soils with relatively high content of metallic elements should include combined approach (chemical composition of soils, chemical composition of soil solution, mineralogical transformations, chemical composition of plants). Furthermore, our study showed that research focused on uptake of metallic elements by plants in soils under the influence the soil additives should also consider the nature of specific element and biology of plant species.

### Supplementary Information

Below is the link to the electronic supplementary material.

Supplementary file1 (DOCX 23 KB)

Supplementary file2 (DOC 8767 KB)

Supplementary file3 (DOC 79 KB)

Supplementary file4 (DOC 2762 KB)

## Data Availability

All data generated or analyzed during this study are included in this published article (article and supplementary materials).

## Declarations

All authors have read, understood, and have complied as applicable with the statement on "Ethical responsibilities of Authors" as found in the Instructions for Authors.
