# Supplementary material for: Effect of soil additives on biogeochemistry of ultramafic soils—an experimental approach with Brassica napus L
Source: Environ Monit Assess. 2024 Jul 17;196(8):744. doi: 10.1007/s10661-024-12897-4 (PMC11254991; doi:10.1007/s10661-024-12897-4)
Supplement: Supplementary file 1 — Supplementary file1 (DOCX 23 KB) [file 10661_2024_12897_MOESM1_ESM.docx]

**Effect of soil additives on biogeochemistry of ultramafic soils - an experimental approach with *Brassica napus* L.**

Artur Pędziwiatr^1*^, Jakub Kierczak^2^, Anna Potysz^2^, Anna Pietranik^2^

^1^Warsaw University of Life Sciences, Institute of Agriculture, Department of Soil Science, Nowoursynowska Str. 159, b.37, 02-787 Warszawa, Poland (ORCID: 0000-0002-6253-4302)

^2^University of Wrocław, Faculty of Earth Sciences and Environmental Management, Institute of Geological Sciences, Department of Experimental Petrology, Maxa Borna Str. 9, 50-204 Wrocław, Poland (ORCID: 0000-0002-3243-6832; 0000-0002-7034-367X; 0000-0003-3990-8721)

*corresponding author: artur_pedziwiatr@sggw.edu.pl

**Table 1.** Air conditions in greenhouse during the experiment.

| Parameter | Value |
| --- | --- |
| Average temperature/day | 24.3 [°C] |
| Average temperature/night | 20.0 [°C] |
| Average temperature/24h | 21.7 [°C] |
| Average Relative Humidity/day | 55.4 [%] |
| Average Relative Humidity/night | 59.7 [%] |
| Average Relative Humidity/24h | 61 [%] |
| Sum of radiation/24h | 199.9 [J/cm˛] |

**Table 2.** Operating conditions during analysis using Inductively Coupled Plasma – Optical Emission Spectrometer Avio 200^TM^ Perkin Elmer (Waltham, Massachusetts, the USA).

| Parameter | Value |
| --- | --- |
| Plasma gas (argon) flow | 12 L/min |
| Auxiliary gas (argon) flow | 0.2 L/min |
| Nebulizer gas (argon) flow | 0.6 L/min |
| RF power | 1500 W |
| Pump flow rate | 1 ml/min |
| Spray chamber: | TFE Tracey Spray Chamber |
| Wavelengths: |  |
| Ca | 317.993 nm |
| Mg | 285.213 nm |
| Na | 589.592 nm |
| K | 766.490 nm |
| P | 214.914 nm |
| Ni | 231.604 nm |
| Cr | 284.325 nm |
| Co | 228.616 nm |
| Mn | 257.610 nm |
| Fe | 259.939 nm |
| Al | 396.153 nm |

**Description 1.** Details of soil buffer properties measurements.

Ttitration curves were obtained by adding an incremental amount of 0.1 M HCl (from 0 cm^3^ up to 10 cm^3^) and 0.1 M NaOH (from 0 cm^3^ up to 10 cm^3^) to soil (10 g, <2 mm) suspension with MiliQ H_2_O (from 10 cm^3^ up to 20 cm^3^). Hence, 10 g of each soil sample was suspended in a polyethylene tube with 20 mL of solution. The blank sample was processed in the same way as the soil samples. The suspensions were equilibrated at 25°C±3°C for 24 h. Afterwards, the pH of the suspensions was measured. The buffer area (in cm^2^) was determined planimetrically.

**Description 2.** Details of the test concerning on the potential of the soil for acid neutralization.

Acid neutralization and acid production capacity were established based on sulfur content in the samples and a back titration experiment. A portion of 10 g of each sample, prepared in duplicate, was placed into Erlenmeyer flasks (250 cm^3^ capacity) which were then filled with 100 cm^3^ of distilled water. First, these samples were equilibrated for 15 min and their pH was measured. Then, back titration with 2N H_2_SO_4_ was performed over the course of 24 h till final pH of 3.5 was reached. Acid neutralizing capacity (atom per formula unit) per ton of soil was calculated based on Eq. 1:

Acid consumption = (amount of acid [cm^3^] x 0.049 x 1000 ) / sample weight [g] Eq.1

Whereas acid production potential was calculated as follows:

Acid production potential = S content [%] x 30.6 Eq.2

The acid production potential value was subtracted from the acid consumption value to determine sample characteristics. A negative value was assumed to indicate the potential of a sample to be acid producing, whereas a positive value was assumed to indicate the potential of a sample to be acid neutralizing.

**Description 3.** Details of the measurements of zero point of charge in soil (pH_0_).

The 0.5 cm^3^ of 0.1 M CaCl_2_ was added to 15 replicates of soil sample (4 g). In beakers numbered from 1 to 7, increasing amounts of 0.1 M HCl were added, whereas for beakers numbered from 9 to 15 increasing amounts of 0.1 M NaOH were added. The beakers were filled with MiliQ H_2_O up to 20 cm^3^ (beaker number 8 was only filled with MiliQ H_2_O). After 4 days, the pH (pH 0.002 M) of each suspension was measured. In the next step, 0.5 cm^3^ of 2 M CaCl_2_ was added to each suspension, and each suspension was agitated for 3 h. The pH of each suspension was then measured (pH 0.05 M), the Δ pH was calculated (pH 0.05 M – pH 0.002 M), and Δ pH versus pH 0.002 M was graphed. Then, pH_0_ was calculated based on the graph (Pansu and Gautheyrou 2006).

**Table 2.** Chemical composition of Metranal™ 34 (former CRM 7004) Quality Control Material.

| Oxide | Expected value | Measured value^1^ |
| --- | --- | --- |
|  | [wt. %] | |
| Al_2_O_3_ | 13.10 | 12.22 ± 2.89 |
| CaO | 2.07 | 1.66 ± 0.06 |
| MgO | 1.29 | 1.22 ± 0.01 |
| Fe_2_O_3_ | 5.82 | 4.93 ± 0.06 |
| K_2_O | 2.55 | 1.73 ± 0.06 |
| Na_2_O | 0.72 | 0.64 ± 0.01 |
| P_2_O_5_ | 0.45 | 0.17 ± <0.01 |
| Element | [mg · kg^-1^] | |
| Ni | 33.1± 1.1 | 32.88 ± 0.68 |
| Cr | 82.2± 6.3 | 76.68 ± 0.75 |
| Co | 20 ± 1.3 | 22.35 ± 0.31 |
| Mn | 869 ± 34 | 773 ± 6 |

^1^Values are presented as mean values from three independent measurements ± standard deviation.

**Table 3.** Chemical composition of NIST Standard Reference Material 1573a® Tomato Leaves.

| Element | Expected value | Measured value^1^ |
| --- | --- | --- |
|  | [mg · kg^-1^ DW] | |
| Al | 598.4 ± 7.1 | 504.87 ± 15.72 |
| Ca | 50450 ± 550 | 47752.1 ± 2309.6 |
| Cr | 1.988 ± 0.034 | 1.34 ± 0.27 |
| Co | 0.5773 ± 0.0071 | 0.44 ± 0.01 |
| Fe | 367.5 ± 4.3 | 304.19 ± 2.86 |
| Mn | 246.3 ± 7.1 | 214.07 ± 1.66 |
| Ni | 1.582 ± 0.041 | 1.56 ±0.06 |
| P | 2161 ± 28 | 1740.4 ± 18.1 |
| K | 26760 ± 480 | 24685.97 ± 368.05 |
| Na | 136.1 ± 3.7 | 159.69 ± 61.52 |
| Mg | not determined | 9985.71 ± 126.11 |

^1^Values are presented as mean values from four independent measurements ± standard deviation.

**Table 4.** Chemical composition of B2186 Soil Standard (Loamy) and B2166 Birch Leaf Standard - Elemental Microanalysis Elemental Lab®.

| Element | Expected value | Measured value^1^ |
| --- | --- | --- |
|  | [wt. %] | |
| B2186 Soil Standard (Loamy) | | |
| C | 2.75±0.12 | 2.71±<0.01 |
| N | 0.27±0.01 | 0.29±<0.01 |
| S | (0.04) | 0.03±<0.01 |
| B2166 Birch Leaf Standard | | |
| C | 48.1±0.51 | 49.0±003 |
| N | 2.12±0.06 | 2.21±<0.01 |
| S | 0.17±0.03 | 0.18±<0.01 |

^1^Values are presented as mean values from two independent measurements ± standard deviation.

**Table 5.** Parameters of XRD analysis using Bruker AXS D5005 diffractometer.

| Parameter | Description/value |
| --- | --- |
| X-ray generator | KRISTALLOFLEX^®^760 |
| Divergence slit | 1 [mm] |
| Anti-scatter slit | 2 [mm] |
| Detector slit | 0.6 [mm] |
| Radiation | CoKα |
| Voltage | 40 kV |
| Current | 35 mA |
| Counting time | 1s for a 0.02° step (the range of angles was from 3° to 70° 2θ) |

**Table 6.** Parameters of SEM (JSM IT-100, JEOL) analysis.

| Parameter | Description/value |
| --- | --- |
| Chamber pressure | High and low vacuum modes |
| Accelerating voltage | 15 kV |
| Detector distance from the sample | 10 mm |
