# Supplementary material for: Effect of soil additives on biogeochemistry of ultramafic soils—an experimental approach with Brassica napus L
Source: Environ Monit Assess. 2024 Jul 17;196(8):744. doi: 10.1007/s10661-024-12897-4 (PMC11254991; doi:10.1007/s10661-024-12897-4)
Supplement: Supplementary file 2 — Supplementary file2 (DOC 8767 KB) [file 10661_2024_12897_MOESM2_ESM.doc]

**Effect of soil additives on biogeochemistry of ultramafic soils - an experimental approach with *Brassica napus* L.**

Artur Pędziwiatr^1*^, Jakub Kierczak^2^, Anna Potysz^2^, Anna Pietranik^2^

^1^Warsaw University of Life Sciences, Institute of Agriculture, Department of Soil Science, Nowoursynowska Str. 159, b.37, 02-787 Warszawa, Poland (ORCID: 0000-0002-6253-4302)

^2^University of Wrocław, Institute of Geological Sciences, Department of Experimental Petrology, Maxa Borna Str. 9, 50-204 Wrocław, Poland (ORCID: 0000-0002-3243-6832; 0000-0002-7034-367X; 0000-0003-3990-8721)

*corresponding author: artur_pedziwiatr@sggw.edu.pl

Figures 1-6. X-ray diffractograms presenting mineralogy of powdered serpentinite and fine earth fraction of ultramafic soils from pot experiment, and mineralogy of clay fraction of these soil (Srp-serpentine, Spl-spinel, Qz-quartz, Fsp-feldspar, Chl-chlorite, Ms-muscovite/mica, Sm-smectite). Abbreviations of minerals are given after Warr (2021).


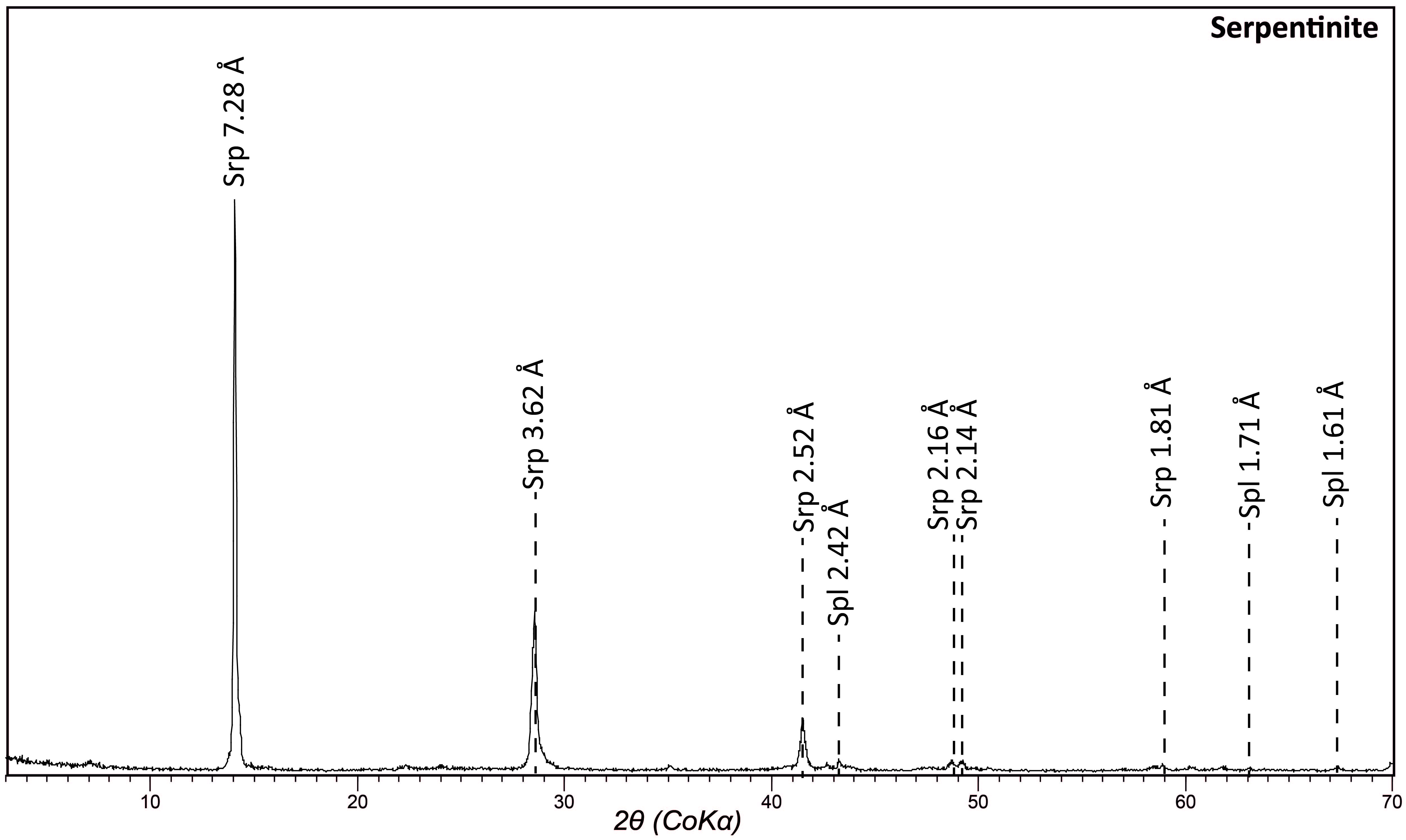


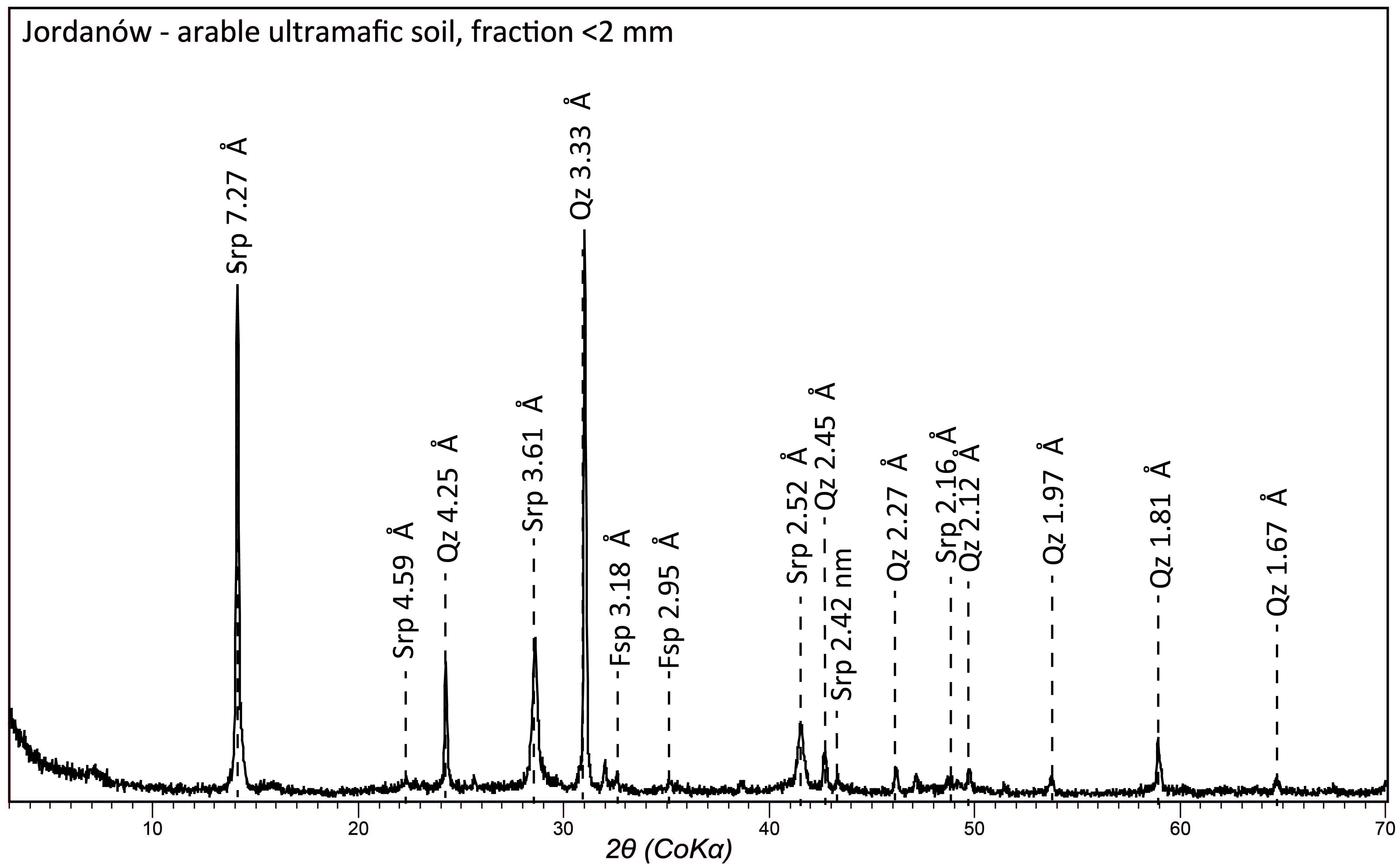


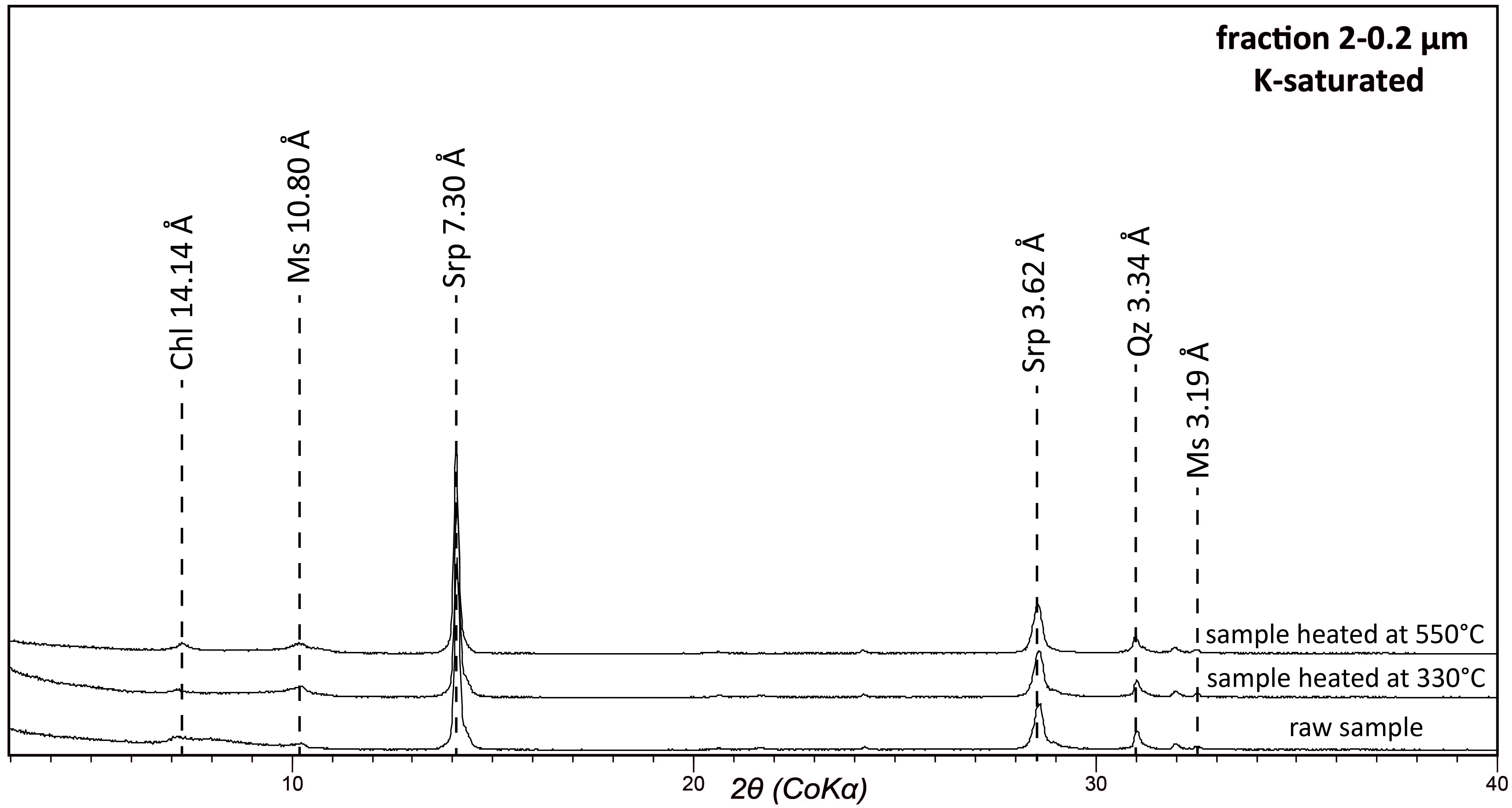


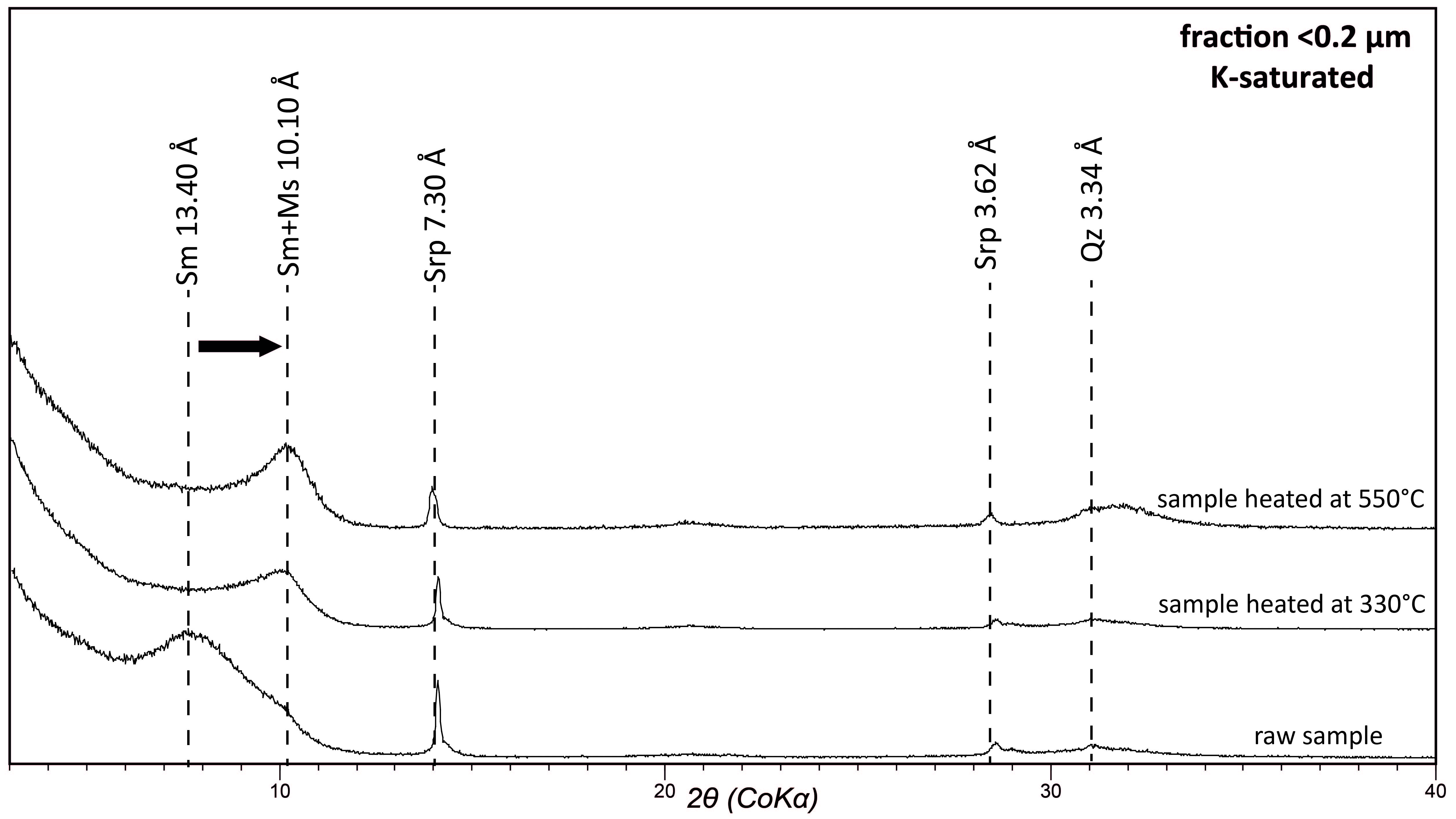


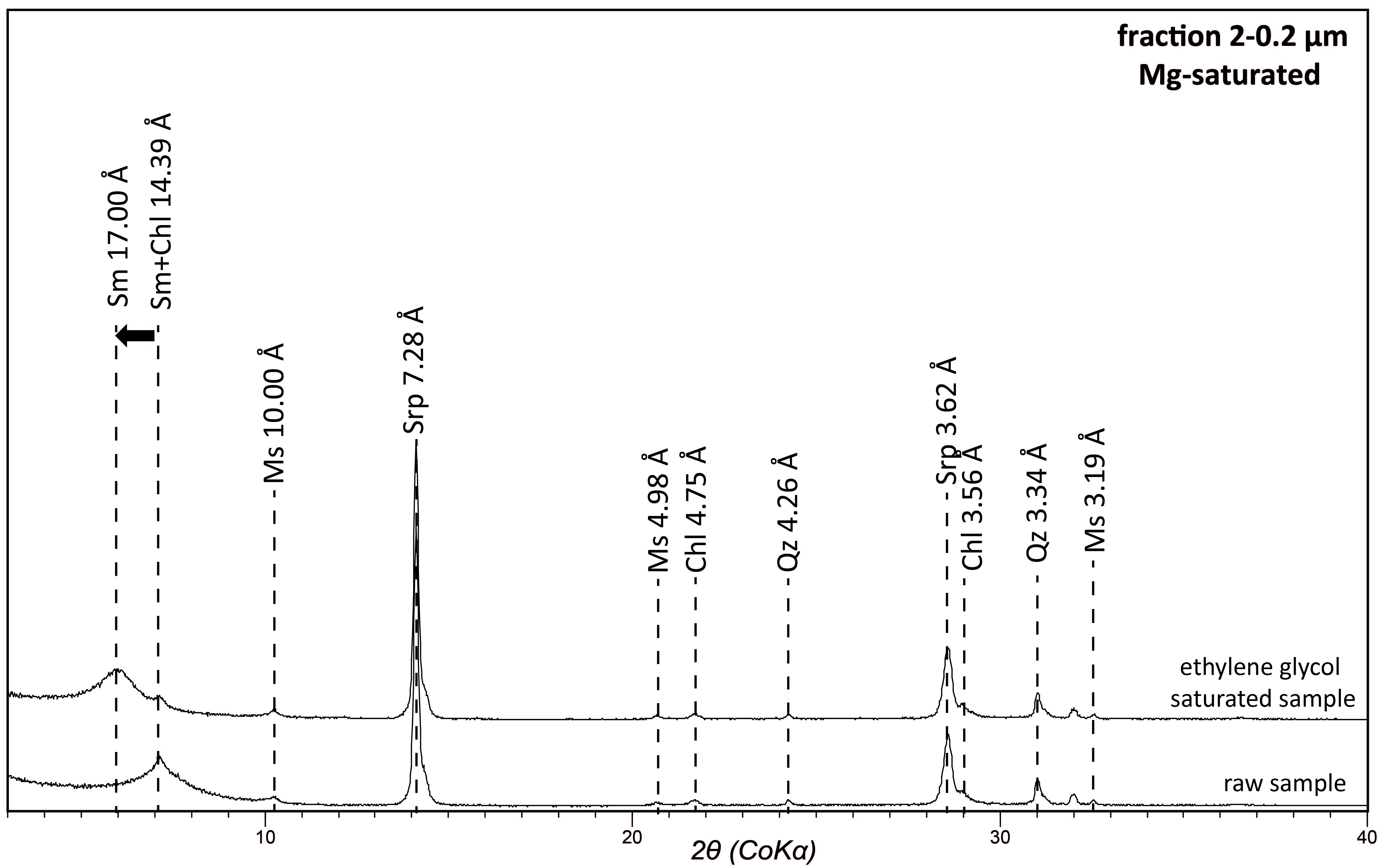


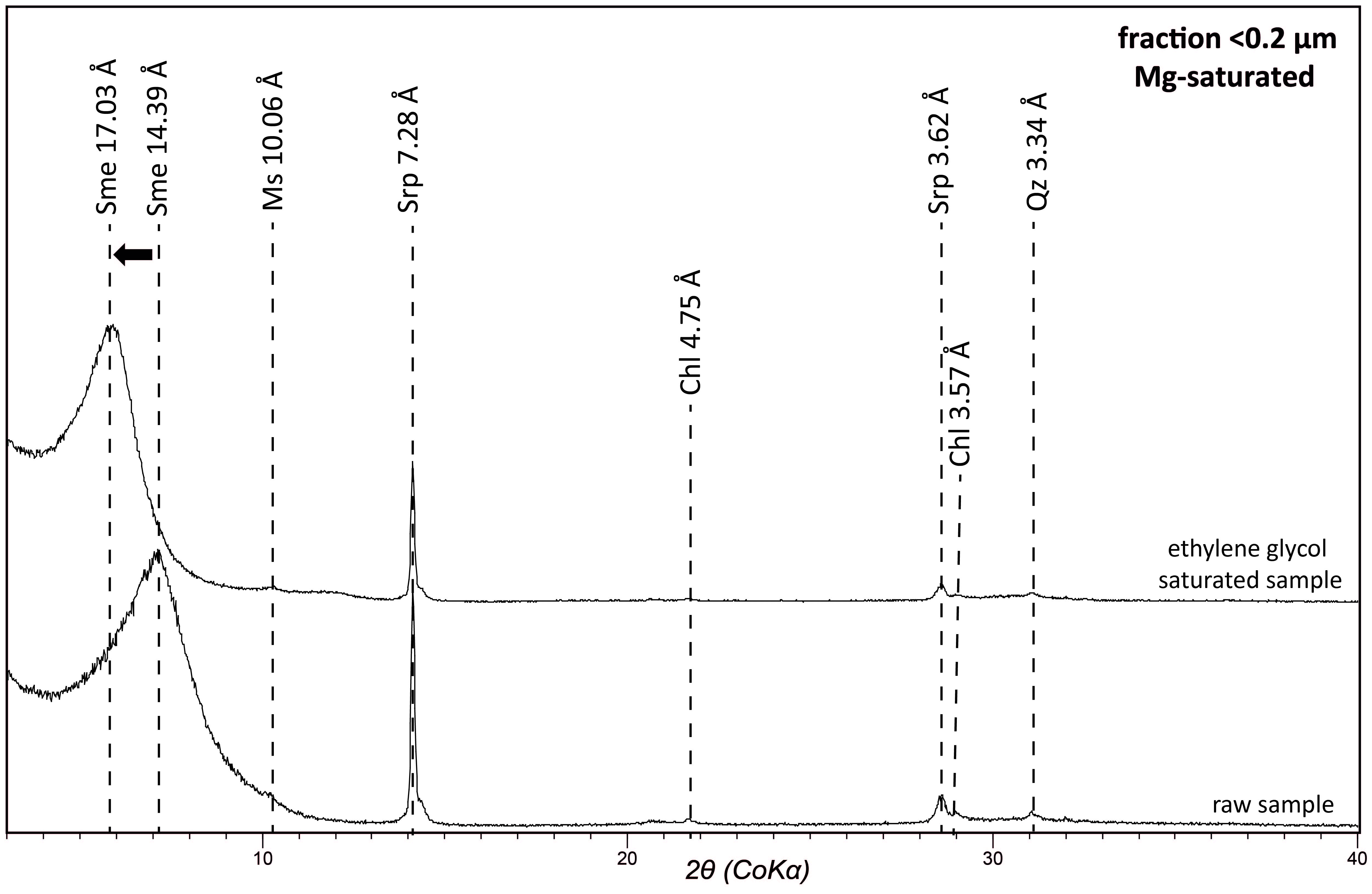


Warr, L.N., 2021. IMA–CNMNC approved mineral symbols. Mineral. Mag. 85, 291–320.
