# Supplementary material for: Effect of soil additives on biogeochemistry of ultramafic soils—an experimental approach with Brassica napus L
Source: Environ Monit Assess. 2024 Jul 17;196(8):744. doi: 10.1007/s10661-024-12897-4 (PMC11254991; doi:10.1007/s10661-024-12897-4)
Supplement: Supplementary file 3 — Supplementary file3 (DOC 79 KB) [file 10661_2024_12897_MOESM3_ESM.doc]

**Effect of soil additives on biogeochemistry of ultramafic soils - an experimental approach with *Brassica napus* L.**

Artur Pędziwiatr^1*^, Jakub Kierczak^2^, Anna Potysz^2^, Anna Pietranik^2^

^1^Warsaw University of Life Sciences, Institute of Agriculture, Department of Soil Science, Nowoursynowska Str. 159, b.37, 02-787 Warszawa, Poland (ORCID: 0000-0002-6253-4302)

^2^University of Wrocław, Faculty of Earth Sciences and Environmental Management, Institute of Geological Sciences, Department of Experimental Petrology, Maxa Borna Str. 9, 50-204 Wrocław, Poland (ORCID: 0000-0002-3243-6832; 0000-0002-7034-367X; 0000-0003-3990-8721)

*corresponding author: artur_pedziwiatr@sggw.edu.pl

Table 1. Nickel, Cr, Co, Ca, Mg, K content, and pH of soil solution collected using MicroRhizon® samplers in the pot experiment filled with ultramafic soil and fertilizers - raw data.

|  | Soil additive | | Ni | Cr | Co | Ca | Mg | K | pH |
| --- | --- | --- | --- | --- | --- | --- | --- | --- | --- |
|  |  | [µg dm^3^] | | | | [mg dm^3^] | | |  |
| *Before sowing* | | | | | | | | | |
|  | T1-control | | 383.74 | 10.67 | 3.83 | 78.30 | 58.51 | 61.81 | 4.91 |
|  | T1-control | | 407.11 | 7.28 | <d.l. | 91.80 | 80.17 | 86.28 | 4.77 |
|  | T1-control | | 386.92 | 9.22 | 2.71 | 63.80 | 55.92 | 67.53 | 4.87 |
|  | T2-manure | | 728.31 | 14.79 | 53.32 | 172.91 | 139.85 | 308.23 | 6.77 |
|  | T2-manure | | 549.60 | 18.34 | 23.21 | 125.94 | 95.08 | 244.97 | 6.59 |
|  | T2-manure | | 697.98 | 21.91 | 39.50 | 136.83 | 111.79 | 270.56 | 6.78 |
|  | T3-rosahumus | | 463.12 | 10.63 | 2.13 | 71.13 | 68.73 | 84.00 | 4.92 |
|  | T3-rosahumus | | 418.20 | 14.50 | 5.62 | 54.38 | 50.63 | 72.43 | 5.13 |
|  | T3-rosahumus | | 394.36 | 12.65 | 6.66 | 52.07 | 46.15 | 63.02 | 5.09 |
|  | T4-KNO_3_ | | 532.91 | 6.40 | 3.56 | 108.93 | 102.13 | 131.57 | 4.66 |
|  | T4-KNO_3_ | | 558.19 | 11.43 | 4.37 | 101.81 | 84.50 | 114.03 | 4.77 |
|  | T4-KNO_3_ | | 643.69 | 8.60 | 6.62 | 119.91 | 113.90 | 109.92 | 4.93 |
|  | T5-lime | | 218.90 | 12.25 | 3.94 | 101.37 | 48.25 | 48.87 | 7.61 |
|  | T5-lime | | 192.94 | 11.18 | 5.03 | 180.78 | 70.02 | 63.50 | 7.25 |
|  | T5-lime | | 141.75 | 4.69 | 0.99 | 124.06 | 52.79 | 50.87 | 7.28 |
|  | T6-(NH₄)₂SO₄ | | 2699.89 | 11.94 | 131.45 | 392.45 | 363.34 | 242.87 | 4.36 |
|  | T6-(NH₄)₂SO₄ | | 1946.93 | 10.31 | 71.07 | 316.05 | 253.79 | 199.26 | 4.34 |
|  | T6-(NH₄)₂SO₄ | | 1232.28 | 13.01 | 98.53 | 173.94 | 141.27 | 149.50 | 4.61 |
|  | T7-Ca(H₂PO₄)₂ | | 642.48 | 17.03 | 10.79 | 104.67 | 90.20 | 78.18 | 4.58 |
|  | T7-Ca(H₂PO₄)₂ | | 698.85 | 16.36 | 15.47 | 111.57 | 97.20 | 77.84 | 4.79 |
|  | T7-Ca(H₂PO₄)₂ | | 733.90 | 16.74 | 16.57 | 120.34 | 106.89 | 92.26 | 4.60 |
| *Before harvesting* | | | | | | | | | |
|  | T1-control | | 339.62 | 32.00 | 3.77 | 2.33 | 5.88 | 13.71 | 5.52 |
|  | T1-control | | 298.37 | 23.19 | 1.75 | 0.10 | 3.76 | 15.03 | 5.59 |
|  | T1-control | | 323.29 | 31.17 | 2.65 | 3.17 | 7.07 | 14.16 | 5.49 |
|  | T2-manure | | 214.34 | 7.40 | 8.81 | 6.91 | 6.34 | 22.29 | 6.56 |
|  | T2-manure | | 464.61 | 12.80 | 22.89 | 27.54 | 26.13 | 59.11 | 6.32 |
|  | T2-manure | | 460.27 | 12.58 | 22.05 | 27.56 | 26.61 | 59.36 | 6.30 |
|  | T3-rosahumus | | 541.06 | 52.70 | 4.85 | 3.79 | 6.11 | 14.93 | 5.70 |
|  | T3-rosahumus | | 402.77 | 29.59 | 6.99 | 8.22 | 11.74 | 36.89 | 5.68 |
|  | T3-rosahumus | | 406.98 | 30.40 | 7.30 | 8.49 | 12.31 | 35.99 | 5.65 |
|  | T4-KNO_3_ | | 294.19 | 28.09 | 1.92 | 0.46 | 4.23 | 16.78 | 5.53 |
|  | T4-KNO_3_ | | 262.47 | 24.89 | 3.90 | <d.l. | 2.31 | 5.44 | 5.59 |
|  | T4-KNO_3_ | | 291.31 | 35.93 | 2.69 | 0.95 | 3.58 | 7.64 | 5.79 |
|  | T5-lime | | 125.90 | 3.74 | 3.15 | 65.92 | 20.69 | 27.22 | 7.42 |
|  | T5-lime | | 190.19 | 10.97 | 4.14 | 25.53 | 12.42 | 9.90 | 7.36 |
|  | T5-lime | | 414.86 | 25.34 | 14.34 | 61.75 | 23.51 | 11.93 | 7.34 |
|  | T6-(NH₄)₂SO₄ | | 849.64 | 6.67 | 138.76 | 102.36 | 100.13 | 33.00 | 4.61 |
|  | T6-(NH₄)₂SO₄ | | 2012.48 | 14.31 | 265.46 | 180.93 | 151.98 | 36.49 | 3.91 |
|  | T6-(NH₄)₂SO₄ | | 2932.88 | 16.99 | 493.68 | 249.70 | 222.41 | 78.74 | 3.94 |
|  | T7-Ca(H₂PO₄)₂ | | 369.06 | 27.37 | 4.66 | 2.83 | 6.35 | 6.24 | 5.70 |
|  | T7-Ca(H₂PO₄)₂ | | 347.82 | 33.99 | 2.37 | 1.19 | 4.52 | 8.34 | 5.62 |
|  | T7-Ca(H₂PO₄)₂ | | 464.14 | 33.22 | 4.09 | 8.75 | 11.64 | 15.01 | 5.40 |
