# Supplementary material for: Effect of soil additives on biogeochemistry of ultramafic soils—an experimental approach with Brassica napus L
Source: Environ Monit Assess. 2024 Jul 17;196(8):744. doi: 10.1007/s10661-024-12897-4 (PMC11254991; doi:10.1007/s10661-024-12897-4)
Supplement: Supplementary file 4 — Supplementary file4 (DOC 2762 KB) [file 10661_2024_12897_MOESM4_ESM.doc]

**Effect of soil additives on biogeochemistry of ultramafic soils - an experimental approach with *Brassica napus* L.**

Artur Pędziwiatr^1*^, Jakub Kierczak^2^, Anna Potysz^2^, Anna Pietranik^2^

^1^Warsaw University of Life Sciences, Institute of Agriculture, Department of Soil Science, Nowoursynowska Str. 159, b.37, 02-787 Warszawa, Poland (ORCID: 0000-0002-6253-4302)

^2^University of Wrocław, Faculty of Earth Sciences and Environmental Management, Institute of Geological Sciences, Department of Experimental Petrology, Maxa Borna Str. 9, 50-204 Wrocław, Poland (ORCID: 0000-0002-3243-6832; 0000-0002-7034-367X; 0000-0003-3990-8721)

*corresponding author: artur_pedziwiatr@sggw.edu.pl

**Table. 1.** Chemical composition of *Brassica napus* (aboveground parts and underground parts) cultivated in the pots filled with ultramafic soils and fertilizers.

|  | | Treatment | | Biomass | | | | Ca | | | | Mg | | | Na | | | K | | | P | | | Fe | | | | Mn | | | Ni | | | Cr | | | Co | | | Al | | |  | | |
| --- | --- | --- | --- | --- | --- | --- | --- | --- | --- | --- | --- | --- | --- | --- | --- | --- | --- | --- | --- | --- | --- | --- | --- | --- | --- | --- | --- | --- | --- | --- | --- | --- | --- | --- | --- | --- | --- | --- | --- | --- | --- | --- | --- | --- | --- |
|  |  |  | | | [g] | | | | [% DW] | | | | | | | | | | | | | | | | [mg kg^-1^ DW] | | | | | | | | | | | | | | | | | | | |  |
| *Aboveground parts* | | | | | | | | | | | | | | | | | | | | | | | | | | | | | | | | | | | | | | | | | | | |  | |
|  | T1-control | | 2.30 | | | | 1.37 | | | | 0.76 | | | 0.13 | | | 3.89 | | | 0.25 | | | 94.82 | | | | 46.81 | | | 26.90 | | | 3.93 | | | 0.65 | | | 32.09 | | |  | | | |
|  | T1-control | | 1.69 | | | | 1.38 | | | | 0.68 | | | 0.08 | | | 3.59 | | | 0.26 | | | 73.04 | | | | 36.83 | | | 24.83 | | | 2.74 | | | 0.57 | | | 26.53 | | |  | | | |
|  | T1-control | | 2.14 | | | | 1.29 | | | | 0.72 | | | 0.06 | | | 3.79 | | | 0.21 | | | 104.8 | | | | 49.73 | | | 28.27 | | | 3.94 | | | 0.88 | | | 44.58 | | |  | | | |
|  | T2-manure | | 7.54 | | | | 1.92 | | | | 0.77 | | | 0.40 | | | 5.30 | | | 0.42 | | | 110.0 | | | | 51.14 | | | 14.44 | | | 2.86 | | | 0.53 | | | 37.94 | | |  | | | |
|  | T2-manure | | 6.18 | | | | 2.14 | | | | 0.92 | | | 0.50 | | | 5.52 | | | 0.37 | | | 131.7 | | | | 54.32 | | | 15.98 | | | 3.13 | | | 0.61 | | | 45.93 | | |  | | | |
|  | T2-manure | | 6.76 | | | | 2.20 | | | | 0.80 | | | 0.42 | | | 5.38 | | | 0.43 | | | 99.09 | | | | 57.56 | | | 15.88 | | | 2.77 | | | 0.50 | | | 26.72 | | |  | | | |
|  | T3-rosahumus | | 2.32 | | | | 1.25 | | | | 0.63 | | | 0.05 | | | 3.64 | | | 0.33 | | | 92.10 | | | | 45.91 | | | 21.67 | | | 3.90 | | | 0.55 | | | 38.93 | | |  | | | |
|  | T3-rosahumus | | 1.58 | | | | 1.33 | | | | 0.71 | | | 0.04 | | | 3.82 | | | 0.28 | | | 102.1 | | | | 42.10 | | | 20.72 | | | 2.24 | | | 0.54 | | | 44.04 | | |  | | | |
|  | T3-rosahumus | | 2.14 | | | | 1.38 | | | | 0.72 | | | 0.04 | | | 3.91 | | | 0.32 | | | 175.2 | | | | 54.58 | | | 24.15 | | | 4.06 | | | 0.81 | | | 89.81 | | |  | | | |
|  | T4-KNO_3_ | | 2.55 | | | | 1.29 | | | | 0.68 | | | 0.07 | | | 3.77 | | | 0.26 | | | 87.68 | | | | 45.04 | | | 24.19 | | | 2.82 | | | 0.62 | | | 27.63 | | |  | | | |
|  | T4-KNO_3_ | | 2.95 | | | | 1.20 | | | | 0.61 | | | 0.04 | | | 3.97 | | | 0.33 | | | 100.4 | | | | 56.83 | | | 28.47 | | | 3.47 | | | 0.72 | | | 36.60 | | |  | | | |
|  | T4-KNO_3_ | | 2.44 | | | | 1.22 | | | | 0.62 | | | 0.06 | | | 3.34 | | | 0.27 | | | 94.54 | | | | 46.20 | | | 27.79 | | | 3.72 | | | 0.66 | | | 35.50 | | |  | | | |
|  | T5-lime | | 2.39 | | | | 3.35 | | | | 0.66 | | | 0.15 | | | 5.35 | | | 0.38 | | | 134.5 | | | | 39.71 | | | 9.50 | | | 4.06 | | | 0.58 | | | 34.28 | | |  | | | |
|  | T5-lime | | 1.87 | | | | 3.12 | | | | 0.67 | | | 0.15 | | | 3.84 | | | 0.32 | | | 182.9 | | | | 41.14 | | | 10.95 | | | 4.74 | | | 0.65 | | | 66.53 | | |  | | | |
|  | T5-lime | | 4.40 | | | | 3.10 | | | | 0.65 | | | 0.13 | | | 4.45 | | | 0.34 | | | 157.1 | | | | 42.37 | | | 11.87 | | | 4.41 | | | 0.79 | | | 61.70 | | |  | | | |
|  | T6-(NH₄)₂SO₄ | | 2.66 | | | | 1.62 | | | | 0.86 | | | 0.19 | | | 6.32 | | | 0.27 | | | 145.0 | | | | 495.4 | | | 86.21 | | | 3.75 | | | 11.51 | | | 57.92 | | |  | | | |
|  | T6-(NH₄)₂SO₄ | | 3.55 | | | | 1.36 | | | | 0.83 | | | 0.15 | | | 7.15 | | | 0.24 | | | 112.5 | | | | 430.9 | | | 85.36 | | | 2.85 | | | 11.77 | | | 47.32 | | |  | | | |
|  | T6-(NH₄)₂SO₄ | | 3.46 | | | | 1.51 | | | | 0.81 | | | 0.20 | | | 6.41 | | | 0.27 | | | 170.7 | | | | 571.9 | | | 86.05 | | | 3.73 | | | 14.95 | | | 59.78 | | |  | | | |
|  | T7-Ca(H₂PO₄)₂ | | 2.37 | | | | 1.30 | | | | 0.60 | | | 0.04 | | | 3.08 | | | 0.28 | | | 80.07 | | | | 56.10 | | | 26.62 | | | 2.83 | | | 0.72 | | | 26.72 | | |  | | | |
|  | T7-Ca(H₂PO₄)₂ | | 2.38 | | | | 1.37 | | | | 0.63 | | | 0.05 | | | 2.91 | | | 0.28 | | | 103.5 | | | | 58.83 | | | 26.77 | | | 2.98 | | | 0.72 | | | 36.08 | | |  | | | |
|  | T7-Ca(H₂PO₄)₂ | | 2.00 | | | | 1.37 | | | | 0.68 | | | 0.07 | | | 2.96 | | | 0.22 | | | 74.45 | | | | 50.16 | | | 24.15 | | | 2.44 | | | 0.66 | | | 21.31 | | |  | | | |
| *Underground parts* | | | | | | | | | | | | | | | | | | | | | | | | | | | | | | | | | | | | | | | | | | | |  | |
|  | | T1-control | | | | nd^1^ | | | | 0.46 | | | 0.46 | | | 0.18 | | | 3.18 | | | 0.43 | | | | 1571 | | | 65.19 | | | 95.79 | | | 30.53 | | | 5.31 | | | 963.2 | | | | |
|  | | T1-control | | | | nd | | | | 0.50 | | | 0.60 | | | 0.18 | | | 2.76 | | | 0.45 | | | | 2230 | | | 61.97 | | | 107.9 | | | 42.04 | | | 5.08 | | | 1170 | | | | |
|  | | T1-control | | | | nd | | | | 0.74 | | | 0.68 | | | 0.17 | | | 2.55 | | | 0.45 | | | | 3055 | | | 119.6 | | | 137.1 | | | 65.58 | | | 8.32 | | | 1424 | | | | |
|  | | T2-manure | | | | nd | | | | 0.84 | | | 0.56 | | | 0.22 | | | 3.45 | | | 0.52 | | | | 2464 | | | 77.16 | | | 99.90 | | | 63.07 | | | 8.32 | | | 1069 | | | | |
|  | | T2-manure | | | | nd | | | | 1.58 | | | 0.58 | | | 0.34 | | | 3.39 | | | 0.54 | | | | 2080 | | | 117.6 | | | 101.9 | | | 41.76 | | | 8.63 | | | 1138 | | | | |
|  | | T2-manure | | | | nd | | | | 0.82 | | | 0.63 | | | 0.28 | | | 4.03 | | | 0.45 | | | | 2095 | | | 98.00 | | | 120.1 | | | 35.28 | | | 12.16 | | | 1231 | | | | |
|  | | T3-rosahumus | | | | nd | | | | 0.44 | | | 0.39 | | | 0.07 | | | 2.97 | | | 0.53 | | | | 867 | | | 32.00 | | | 51.84 | | | 14.32 | | | 2.16 | | | 521.7 | | | | |
|  | | T3-rosahumus | | | | nd | | | | 0.69 | | | 0.63 | | | 0.10 | | | 3.51 | | | 0.39 | | | | 2054 | | | 64.96 | | | 87.77 | | | 93.90 | | | 4.11 | | | 882.6 | | | | |
|  | | T3-rosahumus | | | | nd | | | | 0.62 | | | 0.83 | | | 0.08 | | | 2.99 | | | 0.41 | | | | 4314 | | | 89.68 | | | 154.7 | | | 108.2 | | | 8.71 | | | 2201 | | | | |
|  | | T4-KNO_3_ | | | | nd | | | | 0.50 | | | 0.53 | | | 0.17 | | | 2.79 | | | 0.43 | | | | 1750 | | | 54.18 | | | 94.22 | | | 33.52 | | | 3.77 | | | 876.6 | | | | |
|  | | T4-KNO_3_ | | | | nd | | | | 0.47 | | | 0.66 | | | 0.07 | | | 2.82 | | | 0.41 | | | | 3286 | | | 64.89 | | | 147.0 | | | 65.96 | | | 7.20 | | | 1897 | | | | |
|  | | T4-KNO_3_ | | | | nd | | | | 0.47 | | | 0.56 | | | 0.09 | | | 2.91 | | | 0.54 | | | | 1783 | | | 49.57 | | | 89.99 | | | 35.33 | | | 4.23 | | | 937.3 | | | | |
|  | | T5-lime | | | | nd | | | | 3.02 | | | 0.58 | | | 0.17 | | | 4.10 | | | 0.40 | | | | 2178 | | | 43.54 | | | 81.24 | | | 44.17 | | | 5.11 | | | 1152 | | | | |
|  | | T5-lime | | | | nd | | | | 1.34 | | | 0.37 | | | 0.14 | | | 2.86 | | | 0.48 | | | | 1225 | | | 32.90 | | | 45.87 | | | 20.86 | | | 3.23 | | | 602.7 | | | | |
|  | | T5-lime | | | | nd | | | | 1.05 | | | 0.56 | | | 0.14 | | | 3.64 | | | 0.41 | | | | 2089 | | | 41.28 | | | 73.33 | | | 48.17 | | | 6.16 | | | 1031 | | | | |
|  | | T6-(NH₄)₂SO₄ | | | | nd | | | | 0.41 | | | 0.63 | | | 0.21 | | | 3.58 | | | 0.24 | | | | 2158 | | | 425.2 | | | 193.64 | | | 41.56 | | | 43.19 | | | 1878 | | | | |
|  | | T6-(NH₄)₂SO₄ | | | | nd | | | | 0.48 | | | 0.76 | | | 0.20 | | | 3.55 | | | 0.28 | | | | 3252 | | | 454.2 | | | 244.1 | | | 75.64 | | | 78.38 | | | 2973 | | | | |
|  | | T6-(NH₄)₂SO₄ | | | | nd | | | | 0.44 | | | 0.55 | | | 0.23 | | | 3.64 | | | 0.22 | | | | 2128 | | | 230.3 | | | 146.8 | | | 44.30 | | | 33.47 | | | 1699 | | | | |
|  | | T7-Ca(H₂PO₄)₂ | | | | nd | | | | 0.44 | | | 0.60 | | | 0.12 | | | 2.91 | | | 0.56 | | | | 2259 | | | 57.99 | | | 102.0 | | | 44.13 | | | 4.58 | | | 1218 | | | | |
|  | | T7-Ca(H₂PO₄)₂ | | | | nd | | | | 0.44 | | | 0.61 | | | 0.12 | | | 2.50 | | | 0.47 | | | | 2047 | | | 52.81 | | | 88.47 | | | 47.16 | | | 4.44 | | | 1064 | | | | |
|  | | T7-Ca(H₂PO₄)₂ | | | | nd | | | | 0.48 | | | 0.43 | | | 0.19 | | | 2.69 | | | 0.50 | | | | 1128 | | | 55.83 | | | 74.77 | | | 16.93 | | | 3.57 | | | 593.3 | | | | |

^1^ not determined

**Table. 2.** Content of C, N, and S in aboveground parts of *Brassica napus* cultivated in the pots filled with ultramafic soils and fertilizers.

|  | Treatment | C | N | S |
| --- | --- | --- | --- | --- |
|  |  | [% DW] | | |
|  | T1-control | 41.97 | 3.28 | 0.35 |
|  | T1-control | 41.92 | 2.73 | 0.34 |
|  | T1-control | 41.45 | 2.81 | 0.34 |
|  | T2-manure | 38.46 | 4.73 | 0.83 |
|  | T2-manure | 36.52 | 5.60 | 1.01 |
|  | T2-manure | 36.59 | 5.62 | 1.18 |
|  | T3-rosahumus | 40.99 | 1.96 | 0.41 |
|  | T3-rosahumus | 41.69 | 2.65 | 0.39 |
|  | T3-rosahumus | 41.25 | 2.43 | 0.39 |
|  | T4-KNO_3_ | 41.66 | 2.59 | 0.37 |
|  | T4-KNO_3_ | 41.35 | 2.20 | 0.31 |
|  | T4-KNO_3_ | 41.32 | 1.84 | 0.31 |
|  | T5-lime | 37.80 | 5.87 | 0.73 |
|  | T5-lime | 39.18 | 4.07 | 0.90 |
|  | T5-lime | 38.95 | 4.24 | 0.84 |
|  | T6-(NH₄)₂SO₄ | 36.10 | 6.23 | 1.34 |
|  | T6-(NH₄)₂SO₄ | 36.79 | 6.25 | 1.53 |
|  | T6-(NH₄)₂SO₄ | 34.83 | 6.11 | 1.41 |
|  | T7-Ca(H₂PO₄)₂ | 40.51 | 1.72 | 0.83 |
|  | T7-Ca(H₂PO₄)₂ | 40.74 | 1.82 | 0.87 |
|  | T7-Ca(H₂PO₄)₂ | 41.16 | 2.05 | 0.81 |

**Table. 3.** Nickel, Cr, Co, Mn, Fe, and Al yield of *Brassica napus* cultivated in the pots filled with ultramafic soils and fertilizers (results are presented as mean value from three independent calculations ± standard deviation).

| Treatment | Ni | Cr | Co | Mn | Fe | Al |
| --- | --- | --- | --- | --- | --- | --- |
|  | [mg pot^-1^] | | | | | |
| T1-control | 0.05±0.01 | 0.01±<0.01 | <0.01±<0.01 | 0.09±0.02 | 0.19±0.05 | 0.71±0.21 |
| T2-manure | 0.10±<0.01 | 0.02±<0.01 | <0.01±<0.01 | 0.37±0.02 | 0.77±0.07 | 2.50±0.49 |
| T3-rosahumus | 0.04±0.01 | 0.01±<0.01 | <0.01±<0.01 | 0.10±0.02 | 0.25±0.09 | 1.17±0.54 |
| T4-KNO_3_ | 0.07±0.01 | 0.01±<0.01 | <0.01±<0.01 | 0.13±0.03 | 0.25±0.03 | 0.88±0.15 |
| T5-lime | 0.02±<0.01 | 0.01±<0.01 | <0.01±<0.01 | 0.08±0.01 | 0.31±0.03 | 1.05±0.18 |
| T6-(NH₄)₂SO₄ | 0.28±0.03 | 0.01±<0.01 | 0.04±0.01 | 1.61±0.28 | 0.46±0.09 | 1.76±0.22 |
| T7-Ca(H₂PO₄)₂ | 0.06±0.01 | 0.01±<0.01 | <0.01±<0.01 | 0.12±0.02 | 0.20±0.04 | 0.64±0.18 |


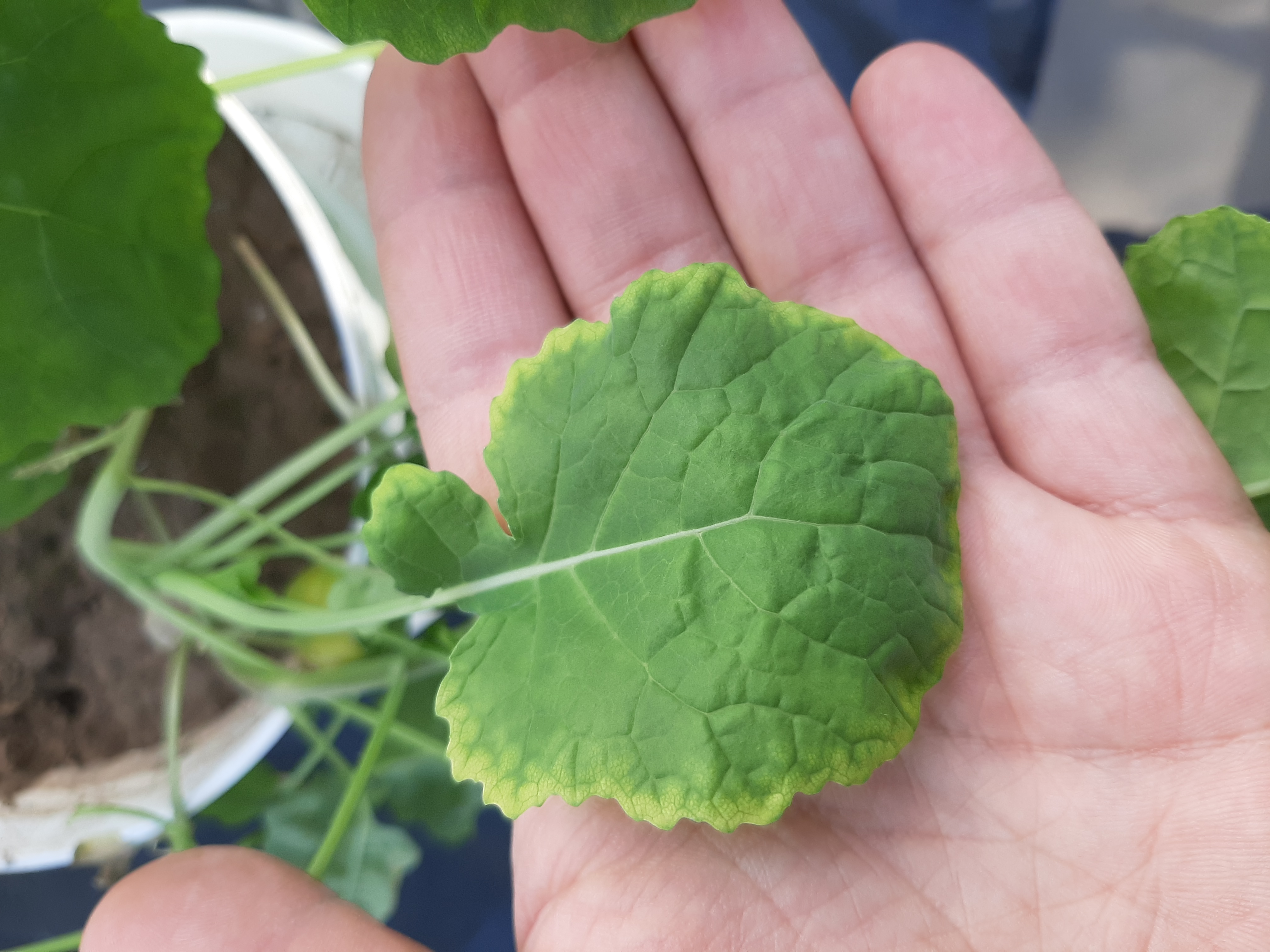


**Figure 1.** Chlorosis observed in *Brasica napus* L. cultivated in fertilized ultramafic soil with (NH₄)₂SO₄.
